# Niche expansion of polyploid cytotypes shaped the phylogeographical history of the *Salix retusa* complex in the European Alpine System

**DOI:** 10.1093/aob/mcaf163

**Published:** 2025-07-25

**Authors:** Loïc Pittet, Piotr Kosiński, Natascha D Wagner, Elvira Hörandl

**Affiliations:** Department of Systematics, Biodiversity, and Evolution of Plants (with Herbarium), University of Göttingen, 37073 Göttingen, Germany; Georg-August University School of Science (GAUSS), University of Göttingen, 37073 Göttingen, Germany; Faculty of Agriculture, Horticulture and Biotechnology, University of Life Sciences, 60-637 Poznań, Poland; Institute of Dendrology, Polish Academy of Sciences, 62-035 Kórnik, Poland; Department of Systematics, Biodiversity, and Evolution of Plants (with Herbarium), University of Göttingen, 37073 Göttingen, Germany; Department of Systematics, Biodiversity, and Evolution of Plants (with Herbarium), University of Göttingen, 37073 Göttingen, Germany

**Keywords:** Abiotic niches, European Alpine System, phylogeography, RAD, *Salix retusa* complex, willows

## Abstract

**Background and Aims:**

Alpine plants exhibit diverse postglacial recolonization patterns following the last glacial periods. Polyploidization may have impacted these dynamics by introducing ecological and physiological novelties that facilitate adaptation to changing environments. However, consistent trends in the recolonization, niche optima and dynamics of polyploids and their related diploids remain elusive. In this study, we investigate the biogeographical history of the *Salix retusa* polyploid complex in the European Alpine System. By comparing genetic patterns and their climatic and edaphic niche optima, we explore how polyploidization shaped species’ geographical distributions by influencing their ecological adaptation.

**Methods:**

RAD sequencing was used to reconstruct the biogeographical history and genetic structure of two related willow species. High-resolution edaphic and climatic data were used to compare the niche optima, breadth and dynamics between the species.

**Key Results:**

The distribution of the polyploid species overlaps with more peripheral refugial areas, which correlates with its broader geographical range in the European Alpine System. However, genetic analyses suggest more potential peripheral glacial refugia within the Alps for the diploid. Our findings indicate niche conservatism within the *S. retusa* complex, with the polyploid species having a broader niche but the diploid being adapted to a more extreme niche.

**Conclusions:**

In our study species, polyploidy is associated with a higher genetic diversity and geographical structure, which might be due to a broader ecological niche and distribution. However, it did not appear to facilitate adaptation or confer a survival advantage during the last glaciation.

## INTRODUCTION

Historical processes such as the Quaternary climate oscillations have impacted not only the geographical distribution of many plant species but also their population size and genetic structure (Comes and Kadereit, 1998; Hewitt, 2000). Landscape heterogeneity as well as species-specific features such as ecological preferences or dispersal ability influence species’ responses to climatic fluctuations (Hewitt, 2004). Although plant species show diverse reactions to Quaternary climate oscillations, previous studies have revealed consistent tendencies in demographic responses and range shifts, particularly among temperate species. In Europe, the phylogeographical patterns for temperate plants suggest that they survived in three major glacial refugia: the Iberian Peninsula, the Apennine Peninsula and the Balkan Peninsula (Taberlet and Cheddadi, 2002). In contrast to temperate lowland species, the diversity of cold-adapted alpine plants and their habitats make it difficult to draw general conclusions about their responses to the last glaciation periods. Evidence for refugia at the periphery of the Last Glaciation Maximum (LGM) ice shield and nunatak refugia has been repeatedly identified in the European Alps (Stehlik *et al*., 2001; Tribsch and Schönswetter, 2003; Schönswetter *et al*., 2005).

Polyploidy is an important factor in the evolution and biogeography of most plant lineages (Soltis *et al*., 2015). Ecological and physiological novelties created by unique gene combinations may have facilitated the adaptation of polyploids to changing environments (Van de Peer *et al*., 2020), and thus polyploidization may have played an important role in past glaciations. Polyploid species often exhibit greater genetic diversity, which may contribute to their enhanced tolerance of biotic and abiotic stress factors and facilitate survival under harsh conditions (Parisod and Broennimann, 2016; Van de Peer *et al*., 2020). In general, polyploids tend to occur in colder climates (Rice *et al*., 2019), and several polyploid complexes have diversified in the high mountain systems of Europe (Kadereit and Abbott, 2021). Polyploidization also allows sympatric speciation due to the formation of rapid reproductive isolation with the progenitors (Otto and Whitton, 2000*b*). Minority cytotype exclusion (Levin, 1975) and competition with progenitor species could be mitigated by a shift in the ecological niche of polyploids (Petit and Thompson, 1999; Theodoridis *et al*., 2013). In small-scale mosaic habitat conditions of alpine vegetation, different cytotypes may show an ecological differentiation in sympatry, as observed in *Jacobaea* (Hülber *et al*., 2015). Other examples suggest patterns of allopatry and niche divergence between diploid and polyploid cytotypes, with the latter often inhabiting the colder areas of the Alps due to their superior cold acclimation (e.g. Tremetsberger *et al*., 2002; Kirchheimer *et al*., 2016; Syngelaki *et al*., 2020; Syngelaki *et al*., 2021; Grünig *et al*., 2024). However, it remains unclear whether polyploids generally tend to higher altitudes, as expected from global temperature gradients (Rice *et al*., 2019). The climatic niche dynamics of diploids and polyploids also lack a consistent trend, with polyploids exhibiting either niche expansions or retractions (Glennon *et al*., 2014).

In addition to climate, abiotic factors such as soil characteristics and topography play a critical role in shaping biogeographical patterns, particularly in mountainous regions. In diploid alpine plants, substrate has been shown to strongly influence spatial genetic structure alongside historical biogeographical processes (Alvarez *et al*., 2009). Yet, despite the importance of these environmental factors, our understanding of niche differentiation between diploids and polyploids, and how this shapes their respective distributions, remains limited, even in lowland plants. One major challenge lies in distinguishing between cytotypes in polyploid complexes, as morphological traits often do not allow for reliable identification. This limitation contributes to a significant gap in our knowledge of polyploid ecology and biogeography (Rosche *et al*., 2025). Molecular markers remain the most effective tool for resolving these species complexes and further identifying intraspecific genetic structure, which captures species’ evolutionary history and demographic processes. Intraspecific genetic structure is often overlooked in ecological studies, potentially limiting our understanding of species’ environmental associations and responses to past changes (Padilla-García *et al*., 2023).

The willow genus (*Salix* L.) comprises 33 species in the European Alps, ∼42 % of which are polyploids (Wagner *et al*., 2021). All *Salix* species are dioecious, which could partially explain the important role of hybridization and polyploidization in the evolutionary history of the genus (Ashman *et al*., 2013). The *Salix retusa* complex includes two closely related taxa, diploid *S. serpillifolia* in the European Alps and polyploid *S. retusa* (incl. *S. kitaibeliana*) in the whole European Alpine System (Büchler, 1986; Dobeš *et al*., 1997; Kosiński *et al*., 2019*a*). This species complex provides an ideal system to investigate the glacial survival of similar species with different ploidy levels, to compare their ecological niche breadths and dynamics, and to infer how polyploidization influences species’ geographical distribution.

We used restriction site associated (RAD) sequencing to address these challenges, as it has proven highly effective in resolving complex phylogenetic and evolutionary questions within *Salix*. It has been recently successfully applied to clarify the phylogeny of European diploid willow species (Wagner *et al*., 2018), to elucidate the evolutionary and phylogenomic relationship of polyploid willow species (Wagner *et al*., 2020, 2021, 2023), and even to reveal the genetic structure of alpine hybrid willow populations (Gramlich *et al*., 2018; Marinček *et al*., 2023; Pittet *et al*., 2025). This method is particularly well-suited for such studies due to its ability to analyse genome-wide genetic variation with high resolution (Eaton and Ree, 2013; Herrera and Shank, 2016).

Here we investigate the genetic structure within *S. retusa* and *S. serpillifolia* including samples from the whole geographical distribution of both species to identify glacial refugia. Genetic groups occurring within the LGM ice shield and exhibiting a high number of private alleles, reduced diversity and low observed heterozygosity were probably subject to bottlenecks and isolation. These groups are interpreted as descendants of nunatak populations (Schorr *et al*., 2012; Schönswetter and Schneeweiss, 2019). Conversely, genetic groups sharing genetic similarities and showing higher levels of genetic diversity probably expanded from a common peripheral refugium (Excoffier *et al*., 2009). To gain insights into whether differential adaptations shaped the observed patterns, we further assess whether *S. serpillifolia* and *S. retusa* have different niche optima and niche breadths in the sympatric area in the Alps. We hypothesize that the species niches are significantly different in the Alps if niche differentiation played a role in polyploid establishment. If the distribution range of *S. retusa* is correlated to a broader ecological niche, its niche breadth would be larger than that of *S. serpillifolia*. Thus, the aims of the study are (1) to elucidate the biogeographical history, (2) to compare the niche optima and dynamics of diploid and polyploid species of the *S. retusa* complex, and (3) to compare the relative impact of these factors on phylogeographical patterns.

## MATERIALS AND METHODS

### Study system and occurrence data

All taxa of the *S. retusa* complex are prostrate dwarf shrubs. They grow in the subalpine and high alpine zones of the European Mountain System, inhabiting grassland communities, screes or even rock crevices.

*Salix retusa s.s.* has different cytotypes from hexaploidy to decaploidy (Kosiński *et al*., 2019*a*). It is the most widespread taxon of the species complex, morphologically very variable and with a broad habitat spectrum. It occurs in most high mountain ranges (Pyrenees, Alps, Apennines, Carpathians, Rila and Dinarids) (Rechinger and Akeroyd, 1964; Skvortsov, 1999; Kosiński *et al*., 2019*a*). *Salix serpillifolia* Scop. is a calcicolous species endemic to the Alps. It has tiny leaves and subsessile catkins, which is an adaptation to high alpine, wind-exposed harsh habitats. Kosiński *et al*. (2019*b*) suggested that it could have survived on nunataks in the Swiss Alps, but sampling in the Eastern Alps remained incomplete in their study. Finally, *S. kitaibeliana* Willd. is sometimes recognized as a separate species or considered a synonym of *S. retusa s.l.* (Hörandl, 1992; Skvortsov, 1999; Kosiński *et al*., 2019*a*). Restricted to the acidic soils of the Tatra Mountains, this taxon is characterized by larger leaves and catkins compared to *S. retusa s.s.* (Kosiński *et al*., 2017). Its limited distribution and the morphological similarity to *S. retusa s.s.* made it difficult to confirm the taxonomic status of this willow without genetic analyses. Moreover, both *S. retusa* and *S. kitaibeliana* are polyploids. Recently, Kosiński *et al*. (2019*a*) found no congruence between morphological patterns and ploidy levels, concluding that *S. kitaibeliana* does not warrant separate taxonomic status and that only *S. retusa s.l.* and *S. serpillifolia* should be recognized as distinct species.

**Fig. 1.**
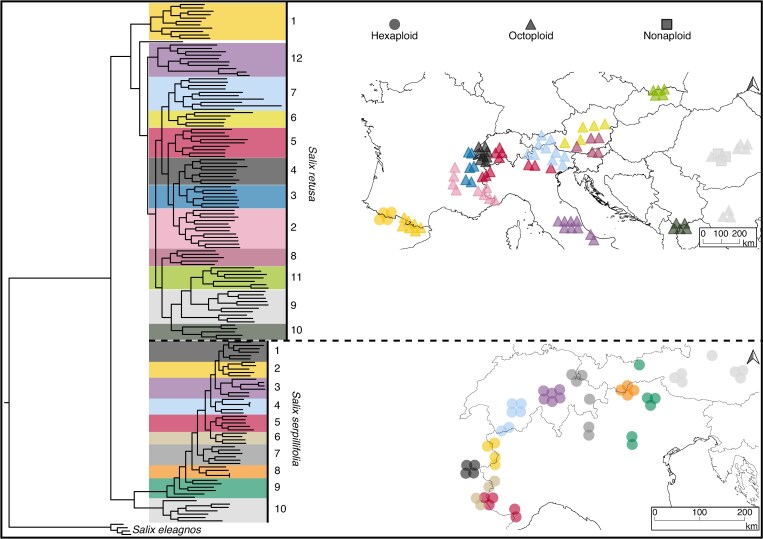
Phylogenetic tree constructed using RAxML analysis based on 38 195 RAD loci (412 147 SNPs; 3 350 514 bp; 41.48 % missing sites), including all individuals of *Salix retusa* and *Salix serpillifolia*, with *Salix eleagnos* as outgroup. Genetic groups (sub-clades) within each species are shown in different colours. These groups are displayed on the accompanying maps using matching colours and were used in subsequent analyses. For *S. retusa*, map symbols additionally reflect ploidy level.

We sampled 100 individuals for *S. retusa s.l.* and 52 individuals for *S. serpillifolia*, covering the whole distribution of both species (see localities in Supplementary Data S1). Sampling followed the strategy of Pittet *et al*. (2025) to cover each geographical area with three to ten samples, depending on the abundance in the respective area. Sampling within the area was mostly random and could not follow a strict scheme as population size is quite variable in small-scale mosaic-like alpine habitats. Leaves were dried in silica gel and herbarium voucher specimens were deposited in the herbarium of the University of Göttingen (GOET). Following the protocol in Wagner *et al*. (2023), we inferred the phylogenetic relationship of all individuals of *S. retusa* and *S. serpillifolia* and confirmed the two species as separate clades by using a RAxML analysis with 38 195 RAD loci [412 147 single nucleotide polymorphisms (SNPs); 3 350 514 bp; 41.48 % missing sites] and *S. eleagnos* as outgroup. We used the phylogenetic sub-clades resulting from the maximum-likelihood (ML) tree to assign individuals to genetic groups within species (Fig. 1). These groups were further used in subsequent analyses (SPAGedi, TreeMix, IBD/IBE). Moreover, precise occurrences of both species across their whole distribution were gathered from the European Vegetation Archive EVA database (Chytrý *et al*., 2016), Infoflora (https://www.infoflora.ch/), GBIF database (https://www.gbif.org/) and our collections. Strong spatial and resolution filtering was applied to the compiled data (for more details on the procedure, see Supplementary Data Appendix S1). Our final observational dataset comprised 779 entries for *S. retusa* and 284 for *S. serpillifolia.*

### Flow cytometry

To complete the chromosome data from the literature (see above) and the comprehensive ploidy level analysis by Kosiński *et al.* (2019*a*) on the species complex, we conducted flow cytometry using silica-gel-dried leaf material to analyse the ploidy levels of all individuals of *S. retusa* and *S. serpillifolia* newly sampled. Using a Tissue Lyzer II (Qiagen; 30 Hz, time 5 s), samples (∼1 cm^2^) were reduced to small pieces and 200 µL of 1 % Otto I extraction buffer was added. Samples were carefully inverted for 1 min before filtering them through CellTrics ® filters (30-µm mesh, Sysmex Partec GmbH, Görlitz, Germany) into flow cytometry sample tubes. To strain the DNA, 800 µL of DAPI-containing Otto II buffer was added (Otto, 1990). Flow cytometry analyses were carried out on a CyFlow Ploidy Analyzer (Sysmex, Norderstedt, Germany) with the Software CUBE v.1.6 (Sysmex, Norderstedt, Germany). The gain was set to 504 nm and three diploid *Salix caprea* samples were used as the external standard. Ploidy level was assessed by the ratio between sample and standard peaks.

### Molecular data generation

DNA of the 156 samples was extracted using the Qiagen DNeasy Plant Mini Kit following the manufacturer’s protocol (Valencia, CA, USA). High-quality DNA was sent to Floragenex, Inc. (Portland, OR, USA) for library preparation following Baird *et al*. (2008) and using the restriction enzyme PstI (Gramlich *et al*., 2018). In polyploids, an increased depth of coverage is necessary to accurately detect and distinguish between the higher number of alleles and improve the reliability of variant detection (Clevenger *et al*., 2018). For this reason, polyploid individuals were sequenced on a separate plate. Quality control of the reads was performed with FastQC v.0.10.1 (Andrews, 2010). After demultiplexing the reads, Stacks v.2.65 (Catchen *et al*., 2013) was used to build *de novo* loci. For both species separately, the key parameters were optimized using a subset of samples and the *r80* method (Paris *et al*., 2017; Rochette and Catchen, 2017). The combination -m 3, -M 4 and -n 4 maximized the number of SNPs and polymorphic loci for the diploid species *S. serpillifolia*. Potential sequencing errors were removed by using a minimum minor allele count of 4 to process an SNP. Additionally, PLINK v.1.90 (Purcell *et al*., 2007) was used to remove individuals with more than 15 % missing genotype rate, sites with more than 10 % missing data and SNPs that deviate from Hardy–Weinberg equilibrium with *P* < 0.01. Only one SNP per locus was retained to avoid linkage disequilibrium.

For the polyploid species *S. retusa*, the parameter combination -m 5, -M 10 and -n 10 in Stacks maximized the number of SNPs and polymorphic loci. Additionally, the maximum number of stacks at a single *de novo* locus was set to 8 because most individuals are octoploid. Genotype calling was done with the R package polyRAD (Clark *et al*., 2019). This package uses the loci catalogue file and the matches file of each individual generated by Stacks, and estimates genotype probabilities from read depth and genetic structure in polyploids. Ploidy levels were assigned to individuals according to flow cytometry results and the literature (Kosiński *et al*., 2019*a*). Loci were filtered out if the total number of individuals with one or more reads for a locus was lower than 80 (80 %) and if the total number of individuals with reads for the minor allele was lower than 4. Non-Mendelian loci were further filtered using the *H*_ind_/*H*_E_ distribution as described in Clark *et al*. (2022). The final dataset was exported in formats compatible with different software that considers ploidy level.

### Genetic structure analysis

The species’ genetic structure was investigated with a principal component analysis (PCA) using the R package adegenet (Jombart and Ahmed, 2011). Additionally, the Bayesian clustering approach implemented in STRUCTURE v.2.3.4 (Pritchard *et al*., 2000) was used to estimate individual ancestry and detect potential admixture. STRUCTURE allows mixed-ploidy data and is more robust than other available methods in such cases (Stift *et al*., 2019). The admixture model was used with correlated allele frequencies and no other prior information. Ten repetitions for each number of genetic clusters (*K*) ranging from 1 to 9 and from 1 to 8 were tested for *S. serpillifolia* and *S. retusa*, respectively. Each run had 50 000 iterations as burn-in, followed by 100 000 Markov chain Monte Carlo (MCMC) iterations. The R package pophelper 2.3.1 (Francis, 2017) was used to estimate the optimal number of clusters *K* according to Evanno *et al*. (2005), and plot the results.

SPAGeDi (Hardy and Vekemans, 2002) was used to compute the average number of alleles per locus (NA), the effective number of alleles per locus (NeA), the rarefied allelic richness (Ark_x_), the gene diversity and the observed heterozygosity (OH). High heterozygosity may reflect postglacial admixture or survival in large, connected refugia, while low heterozygosity can indicate long-term isolation in peripheral refugia or nunataks, where genetic drift and inbreeding are more pronounced. The number of private alleles (alleles unique to a single group) was computed from the R package poppr v.2.9.6 (Kamvar *et al*., 2014). A high number of private alleles can indicate long-term isolation and limited gene flow, as expected in nunataks, whereas fewer private alleles may reflect shared ancestry and gene flow. All statistics were computed for the genetic groups defined by the phylogenetic sub-clades (see Fig. 1) and were tested for significant differences among groups using the Wilcoxon rank-sum test, with Bonferroni correction for multiple comparisons.

To infer gene flow, we used the composite-likelihood approach implemented in TreeMix v.1.13 (Pickrell and Pritchard, 2012). For both species, only biallelic sites were retained and no missing data were allowed to match the analysis requirements. The optimal number of migration events was determined using the Evanno method. Node support was estimated for the ML tree by running 100 bootstrap replicates. The R package BITE v.2 was used to find the optimal number of migration events and for bootstrap analysis (Milanesi *et al*., 2017). Additionally, admixture was tested with the f3-statistic implemented in TreeMix. The tests were made between all triplets of genetic groups within a species, as defined by the phylogenetic sub-clades (see Fig. 1).

### Selection of environmental variables

Environmental variables were divided into two sets representing soil and topographic or climatic conditions. All environmental predictors used for analysis and modelling of species distribution were aggregated (by geographical average) to 250-m spatial resolution and projected to the standard Lambert Azimuthal Equal Area projection for Europe (EPSG:3035).

Soil and topographic variables were obtained from the Global Lithological Map (GLiM, Hartmann and Moosdorf, 2012), SoilGrids (Hengl *et al*., 2017) and GMES RDA project (EU-DEM, www.eea.europa.eu/data-and-maps/data/). GLiM soil lithology was used to generate a bedrock-type layer. The layer was constructed in three steps following Chauvier *et al*. (2021). First, soil lithology was classified into three categories: calcareous, siliceous and mixed. Then, the map was converted to a 100-m grid for each category. Finally, the 100-m grid maps were aggregated to 250-m resolution by averaging proportions of the three categories per 250-m pixel; values range from 0 (only calcareous) to 100 (only siliceous). SoilGrids variables describe topsoil properties that are ecologically relevant for plant species (for details, see Supplementary Data Table S1). The variables elevation, slope and aspect over Europe at 30-m resolution were obtained from EU-DEM. The variable northness was calculated as the cosine of the slope aspect. The variable has no unit but ranges between −1 (south) and 1 (north).

Climatic conditions over the period 1979–2013 were obtained from the Chelsa Climate database (https://chelsa-climate.org) (Karger *et al*., 2017, 2018). The arithmetic means of current climate variables were used to compute 19 bioclimatic layers that were further projected onto a 250 × 250 m grid covering the whole distribution of both species.

For each set of environmental variables, we retained biologically relevant variables with weak correlation (Pearson’s | *r* | < 0.7; Supplementary Data Figs S1 and S2) and variance inflation factor VIF < 5 for niche modelling and comparisons. All variables considered and kept are listed in Table S1.

### Niche modelling and niche comparison

To compare niches, we used the two main approaches described by Guisan *et al*. (2014).

Direct pairwise comparisons of environmental niches were performed between *S. retusa* and *S. serpillifolia.* We compared niches of *S. retusa* and *S. serpillifolia* across their whole respective distribution ranges. We also compared niches of both species in the Alps. Finally, we compared intraspecific niches defined by genetic structures. For *S. retusa*, we compared the niche in the Alps with the niches in the Rila Mountains, Tatra Mountains, South Carpathians, Pyrenees, Apennines, Šar Planina and Korab Massif. For *S. serpillifolia*, we compared the niches in the Western Alps and the Eastern Alps. Direct comparison of niches required species observations and the definition of a background area including suitable habitats. We defined a 20-km buffer zone around the occurrences of each species and sampled 1000 observations per species to generate the background area. For both environmental sets separately, niches were compared following the method described by Broennimann *et al*. (2012) and implemented in the R package ecospat (Di Cola *et al*., 2017). We projected species occurrences onto a two-dimensional grid of 100 × 100 cells derived from PCA. Additionally, kernel smoothing was applied to estimate the density of occurrences of each species in the PCA without overemphasizing outliers or gaps in the data. Using the multivariate space, we compared niche overlap using Schoener’s *D* metric with the correction of the occurrence densities of each species by the prevalence of the environments in their range (Schoener, 1970). A niche equivalency test, where occurrences were pooled and randomly reassigned, was used to assess niche divergence. The process was repeated 1000 times and Schonener’s *D* metric was computed for each repetition. We rejected the null hypothesis of niche equivalency if the observed *D* fell within the lowest 5 % of the computed *D* values. A niche similarity test was used to assess niche conservatism. We tested whether the first niche was more similar to the second niche by randomly selecting pseudo-occurrences from the background area. This process was repeated 1000 times in both directions, and Schonener’s *D* metric was computed for each repetition. If the observed *D* fell within the highest 5 % of the computed *D* values, the niches are more similar than randomly expected. Similarity tests were repeated for each variable separately to identify which variables differ between the niches. The niche change indices for stability, unfilling and expansion were measured as defined by Guisan *et al*. (2014).

Niche comparisons and quantifications based on environmental niche modelling were performed using the R package ENMTools (Warren *et al*., 2010). Comparisons of niche models were performed only between *S. retusa* and *S. serpillifolia* within the Alps. To define the background area for model calibration and evaluation, 1000 pseudo-absence points were randomly generated within a 20-km buffer around known occurrences for each species. Six algorithms, generalized additive models (GAMs), generalized linear models (GLMs), Bioclim (BC), Domain (DM), random forest (RF) and maximum entropy (MaxEnt), were constructed and evaluated using the AUC metric (Nix and Busby, 1986; Carpenter *et al*., 1993; Wood, 2001; Liaw, 2002; Phillips *et al*., 2006). In GAMs, each predictor was modelled using a smooth spline function with a maximum of four basic functions to allow for flexible non-linear relationship while limiting overfitting. GLMs were used with a binomial error distribution. Only models with an AUC > 0.8 were retained. Additionally, model significance was further assessed against a null distribution hypothesis (100 replications) as in Raes and ter Steege (2007). Niche breadth, measured by the metrics *B1* and *B2* (Levins, 1968), and niche overlap, measured by the metric *D* (Schoener, 1970), were used to compare species niche projections. Schoener’s *D* measured the overlap between the environmental niche models (ENMs) of the two species, based on environmental conditions present in the training region (Warren *et al*., 2008).

### Isolation by distance and isolation by environment

To assess the influence of geographical and environmental distances on genetic variation, Mantel tests were performed between genetic distances [*F*_st_/(1 − *F*_st_)], spatial distances (log-transformed Euclidean distance) and environmental distances among genetic groups within a species, as defined by the phylogenetic sub-clades (see Fig. 1). Partial Mantel tests were conducted to examine the relationship between genetic and environmental distances, while controlling for geographical distances, and vice versa. The significance of Mantel tests was evaluated with 10 000 permutations. Environmental distances were calculated by performing a PCA on 16 environmental variables (soil, topography and bioclimatic variables). The first two principal component scores were averaged within each group, and Euclidean distances between groups were computed based on the averaged PCA scores.

To further investigate patterns of isolation by distance and isolation by environment, redundancy analysis (RDA) was performed with individual genotypes (coded as allele counts) as the response variable and geographical coordinates and PCA scores (for the first two environmental components) as explanatory variables. Significance was evaluated with 10 000 permutations. All analyses were conducted using the vegan v.2.6-4 package in R.

## RESULTS

### Flow cytometry

The ploidy level of newly collected *S. retusa* samples varied between hexaploid (Central Pyrenees) and octoploid (Eastern Pyrenees, Alps, Apennines, Šar Planina). Ploidy levels of samples from the Carpathians, Tatra Mountains, Korab Massif and Rila Mountains were previously investigated by Kosiński *et al*. (2019*a*) and ranged from octoploid to decaploid (Supplementary Data S1). For *S. serpillifolia*, all individuals were diploid, confirming previous records (Kosiński *et al*., 2019*a*).

### Population genetic analysis

**Fig. 2.**
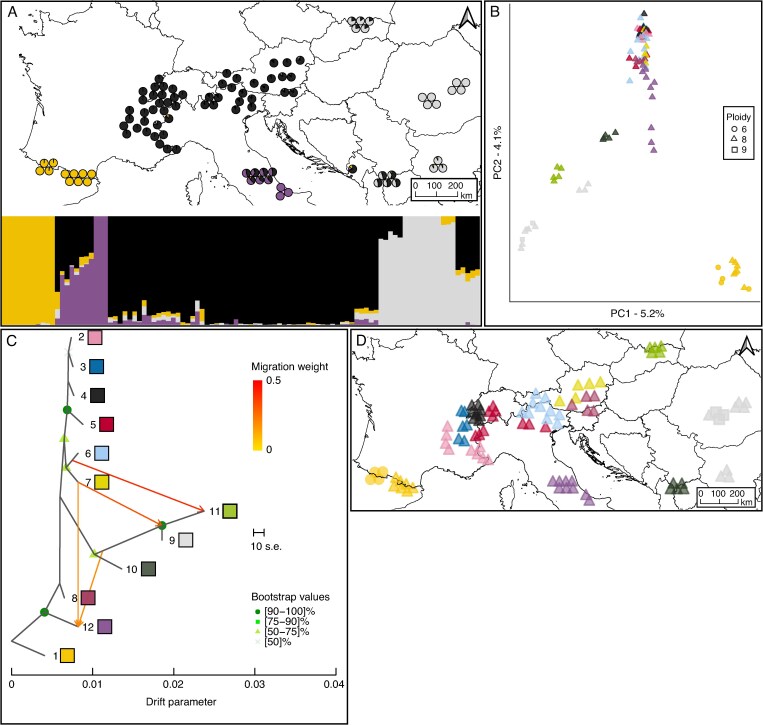
Genetic structure and gene flow between the genetic groups of *Salix retusa* across its distribution range. (A) Map with pie charts and corresponding barplot showing STRUCTURE results for *K* = 4. (B) Principal component analysis of genetic variation among all individuals of *S. retusa*. Each symbol represents an individual, with different symbols indicating ploidy levels and colours corresponding to genetic groups defined by the phylogenetic sub-clades (see Fig. 1). (C) Gene flow between the genetic groups of *S. retusa* as inferred by TreeMix at the most likely number of migrations (m = 4). Arrows indicate the direction of gene flow, with colours representing the percentage of alleles that migrated from the source group. Bootstrap values for the nodes are colour coded. Drift parameter is shown on the *x*-axis. (D) Geographical distribution of the different genetic groups in a map covering the European Alpine System.

RAD sequencing yielded an average of 9.5 × 10^6^ and 8.2 × 10^6^ reads per individual for *S. retusa* and *S. serpillifolia*, respectively. The average quality per read was high, with a minimum Phred score of 36 in all samples. The average depth of read coverage was 56× for *S. retusa* and 48× for *S. serpillifolia.* Based on 44 181 filtered and unlinked loci, STRUCTURE identified four geographically coherent clusters for *S. retusa*, differentiating individuals in the Pyrenees (yellow), across the Alps (black), in the Southern Apennines (purple) and in the easternmost part of the distribution (light grey) (Fig. 2A and Supplementary Data Fig. S3). Additional genetic clusters revealed sub-structure in the Apennines, Carpathians and Rila Mountains (Fig. S4). At *K* = 4, strong admixture was identified in the Tatra Mountains, Central Apennines, Dinaric Alps, Šar Planina and Korab Massif, and Rila Mountains. Most of the genetic structure and admixture patterns were confirmed by the PCA (Fig. 2B). TreeMix identified four migration events, explaining 94.6 % of the variation in the data (Fig. S5). Two signals were oriented from the Eastern Alps (groups 6 and 7) towards the Apennines (group 12), one towards the Carpathians (group 9) and one towards the Tatra Mountains (group 11) (Fig. 2C). A high level of genetic drift was found in the Tatra Mountains, Carpathians and Rila Mountains. The groups in the Alps formed a single clade and showed low levels of genetic drift between each other. The f3-statistic tests did not identify any potential admixture. For *S. retusa*, most private alleles were found in the Pyrenees (706), followed by Apennines (485), and Šar Planina and Korab Massif (258). Rarefied allelic richness was lowest in group 1 (Pyrenees; 2.24 alleles) and highest in group 9 (Carpathians and Rila Mountains; 2.49 alleles). Gene diversity and observed heterozygosity were very similar for all groups, ranging between 0.39 and 0.44 and between 0.33 and 0.37, respectively (Supplementary Data Tables S2–S7). Pairwise *F*_st_ values ranged between 0 and 0.099 (Table S8).

**Fig. 3.**
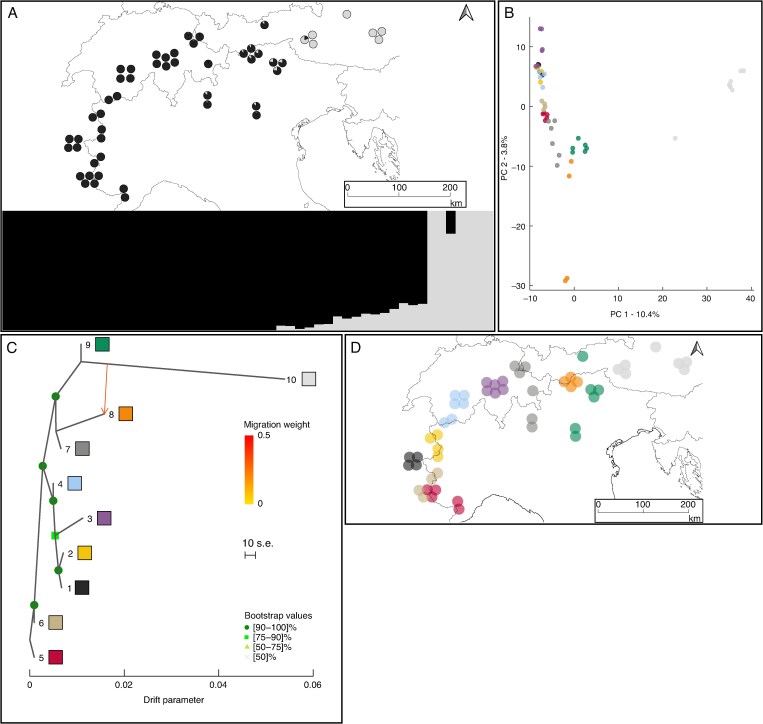
Genetic structure and gene flow between the groups of *Salix serpilllifolia* across its distribution range. (A) Map with pie charts and corresponding barplot showing STRUCTURE results for *K* = 2. (B) Principal component analysis of genetic variation among all individuals of *S. serpillifolia*. Each symbol represents an individual, with colours corresponding to genetic groups defined by the phylogenetic sub-clades (see Fig. 1). (C) Gene flow between the genetic groups of *S. serpillifolia* as inferred by TreeMix at the most likely number of migrations (m = 1). Arrow indicates the direction of gene flow, with colour representing the percentage of alleles that migrated from the source group. Bootstrap values for the nodes are colour coded. Drift parameter is shown on the *x*-axis. (D) Geographical distribution of the different genetic groups in a map covering the European Alps.

Using 28 444 filtered and unlinked loci for *S. serpillifolia*, STRUCTURE identified two distinct genetic clusters, one restricted to the easternmost Alps (westwards to the Hohe Tauern), the other one covering the rest of the Alps (Fig. 3A; Supplementary Data Fig. S6). The model-based genetic structure was confirmed by the PCA (Fig. 3B). One migration event was identified by TreeMix from the easternmost Alps (group 10) to the eastern Central Alps (group 8), under which 99.5 % of the variation in the data was explained (Fig. 3C; Fig. S7). Low levels of genetic drift were found among all groups of the Western Alps, but high levels occurred in the easternmost Alps (group 10). The f3-statistic tests identified groups 6 and 9 as admixed (Table S9). For *S. serpillifolia*, the average number of effective alleles per locus and rarefied allelic richness were similar across all groups, ranging from 1.38 to 1.41 and from 1.22 to 1.24, respectively. Gene diversity ranged from 0.220 to 0.243 and observed heterozygosity ranged from 0.185 to 0.241 (Tables S10–S15). Group 10 (easternmost Alps) was notable for having a high number of private alleles (1348). Pairwise *F*_st_ values ranged between 0.003 and 0.258 (Table S16). Gene diversity and observed heterozygosity were significantly higher in *S. retusa* than in *S. serpillifolia* (Wilcoxon–Mann–Whitney tests: *P* < 0.001).

### Niche modelling and niche comparison

#### Direct niche comparison based on soil and topography

**Fig. 4.**
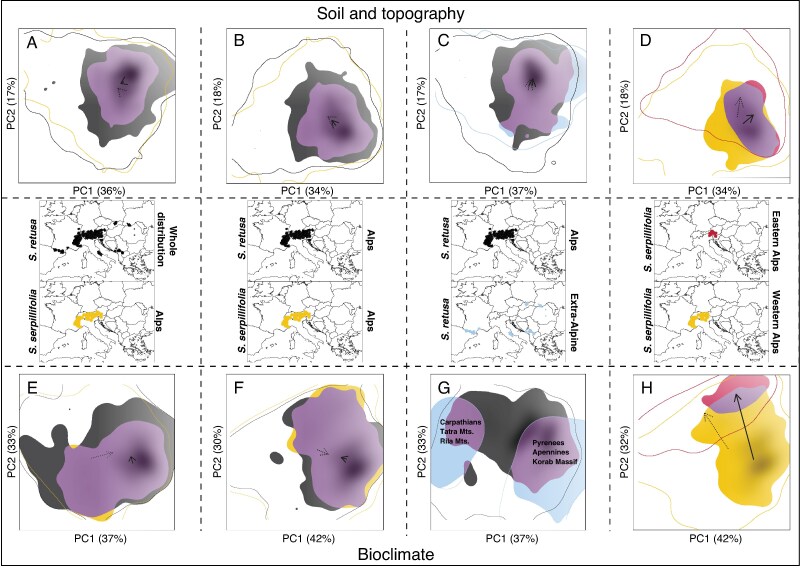
Summary of niche overlaps. Plots of niche overlaps in the two-dimensional space obtained by PCA from the soil and topography variables (A–D) and bioclimatic variables (E–H), between *Salix retusa* and *Salix serpillifolia* across their entire range (A, E), between *S. retusa* and *S. serpillifolia* in the Alps (B, F), between *S. retusa* in the Alps and other mountain ranges (C, G), and between *S. serpillifolia* in the Western and Eastern Alps (D, H). Niche stability is shown in purple, while niche expansion and niche unfilling are represented by the species-specific colours, as indicated on the European maps. Solid contour lines define the extent of environmental conditions within each species’ range. Continuous arrows indicate shifts in niche centroids based on species occurrences. Dotted arrows indicate shifts in niche centroids based on species’ background range.

The first two principal components (PCs) from the PCAs explained an average of 53 % of the variation (Supplementary Data Fig. S8A–D). In these environmental spaces, niche centroids of occurrences and backgrounds were mostly similar, except for the intraspecific level in *S. serpillifolia* (Fig. 4A–D). *Salix retusa* had a broader niche than *S. serpillifolia* across the whole distribution and within the Alps. The niche of *S. retusa* in the Alps was generally broader than outside the Alps. Within *S. serpillifolia*, the western range covered a broader niche than the eastern range. Niche overlap *D* was highest between *S. retusa* in the Alps and *S. serpillifolia,* followed by the overlap between *S. retusa* in and outside the Alps. In contrast, niche overlap was lowest between the western and the eastern ranges of *S. serpillifolia* (Table 1). The niche equivalency and similarity tests indicated that the niches of *S. retusa* and *S. serpillifolia* were less equivalent than expected by chance (*P* = 0.01) but more similar than randomly resampled from the background occurrences (*P* = 0.01 and *P* = 0.02, Table 1). Within both *S. retusa* and *S. serpillifolia*, niche equivalency tests were non-significant (*P* = 0.31 and *P* = 0.73, respectively), but niche conservatism was strongly supported (*P* = 0.03 and *P* = 0.02 for *S.* retusa, *P* = 0.03 and *P* = 0.03 for *S. serpillifolia*; Table 1). Univariate tests of equivalency restricted to the shared environmental conditions revealed that *S. retusa* occupied more north-oriented sites, and sites with a wider range of clay and organic carbon content compared with *S. serpillifolia* (Supplementary Data Table S17, Fig. S9).

**Table 1.** Niche overlap, equivalency and similarity tests results for *Salix retusa* (Sr) and *Salix serpillifolia* (Ss) for both sets of environmental variables, calculated from PCA analyses.

	Soil and topography				Bioclimate			
Species	Sr/Ss	Sr/Ss	Sr	Ss	Sr/Ss	Sr/Ss	Sr	Ss
Range	Entire	Alps	Alps/other ranges	West/East Alps	Entire	Alps	Alps/other ranges	West/East Alps
Schoener’s *D* uncorrected	0.83	0.83	0.64	0.63	0.69	0.74	0.35	0.04
Schoener’s *D* corrected	0.57	0.69	0.61	0.57	0.62	0.76	0.46	0.05
Equivalency (divergence)	0.01	0.01	0.31	0.73	0.48	0.6	1	0.11
Similarity 1–2 (conservatism)	0.03	0.01	0.01	0.03	0.01	0.01	0.02	0.5
Similarity 2–1 (conservatism)	0.01	0.02	0.01	0.03	0.01	0.01	0.04	0.57

#### Niche modelling based on soil and topography

For niche modelling, GAM, GLM, RF and MaxEnt exhibited good performance (AUC > 0.8) and were significantly better than null models (Supplementary Data Table S18). Niche breadth and overlap estimated from these models gave similar results (Table 2). *Salix retusa* had a broader niche than *S. serpillifolia*, with mean values of 0.94 and 0.92, respectively. Niche overlap was about 70 % (Table 2).

**Table 2.** Niche breadth and overlap for *Salix retusa* and *Salix serpillifolia*, based on soil and topography models.

		GAM	GLM	RF	MX
*S. retusa*	Levins’ B1	0.85	0.96	0.97	0.98
	Levins’ B2	0.05	0.3	0.39	0.42
*S. serpillifolia*	Levins’ B1	0.82	0.93	0.94	0.98
	Levins’ B2	0.03	0.13	0.25	0.52
ENMs similarity (Warren *et al*., 2008)	Schoener’s *D*	0.75	0.7	0.64	0.67
	Spearman rank correlation	0.88	0.75	0.57	0.4

#### Direct niche comparison based on bioclimatic variables

The first two principal components of the PCAs accounted for 72 % of the total variance on average (Supplementary Data Fig. S8E–H). As for the soil and topographic data, niche centroids of species distribution were similar between *S. retusa* and *S. serpillifolia* (Fig. 4E, F). However, shifts of the niche centroids of species distribution and background were important at the intraspecific level in both *S. retusa* and *S. serpillifolia* (Fig. 4G, H). When the whole distribution ranges were compared, *S. retusa* had a broader niche than *S. serpillifolia*. However, in the Alps, both species had similar niche breadths. Intraspecific analyses showed that *S. retusa* in the Alps had a broader niche than the extra-Alpine occurrences. *Salix serpillifolia* had a broader niche within the Western Alps than in the Eastern Alps. Niche overlap reached 76 % between *S. retusa* and *S. serpillifolia*, whereas intraspecific overlap was lower (46 % within *S: retusa* and 5 % within *S. serpillifolia*; Table 1). The niches of *S. retusa* and *S. serpillifolia* in the Alps were not less equivalent than expected by chance (*P* = 0.6) but were significantly more similar (*P* = 0.01 for both species; Table 1). Within *S. retusa*, niches were not more divergent (*P* = 1) but were more conserved than expected by chance (*P* = 0.02 and *P* = 0.04, Table 1). For *S. serpillifolia*, niches were neither more divergent (*P* = 0.11) nor more conserved (*P* = 0.5 and *P* = 0.57) than expected by chance (Table 1). According to the univariate tests, *S. retusa* occupied a wider temperature spectrum in the warmest month and in the driest quarter than *S. serpillifolia* (Fig. S9).

#### Niche modelling based on bioclimate

All models exhibited good performance (AUC > 0.8) and were significantly better than null models (Supplementary Data Table S20). Both *S. retusa* and *S. serpillifolia* had broad niches with values ranging from 0.82 to 0.99 according to different models (Table 3). Niche overlap ranged between 47 and 64 %.

**Table 3.** Niche breadth and overlap for *Salix retusa* and *Salix serpillifolia*, based on bioclimatic models.

		GAM	GLM	BC	DM	RF	MX
*S. retusa*	Levins’ B1	0.88	0.95	0.94	0.99	0.96	0.91
	Levins’ B2	0.07	0.29	0.08	0.94	0.33	0.12
*S. serpillifolia*	Levins’ B1	0.82	0.96	0.97	0.99	0.95	0.96
	Levins’ B2	0.03	0.27	0.12	0.96	0.27	0.2
ENMs similarity (Warren *et al*., 2008)	Schoener’s *D*	0.54	0.47	0.64	0.63	0.6	0.58
	Spearman rank correlation	0.76	0.28	0.74	0.54	0.47	0.47

### Isolation by distance and isolation by environment

Mantel tests revealed significant isolation by distance (IBD) for both *S. retusa* (*R*^2^ = 0.56, *P* < 0.001) and *S. serpillifolia* (*R*^2^ = 0.46, *P* = 0.001). No significant contribution of environmental conditions to the genetic structure was found (*R*^2^ = 0.1, *P* = 0.07 for *S. retusa*, *R*^2^ = 0.08, *P* = 0.1 for *S. serpillifolia*). In both species, partial Mantel tests confirmed significant correlations between genetic and spatial distances after controlling for environmental variation (*R*^2^ = 0.53, *P* < 0.001 for *S. retusa*; *R*^2^ = 0.51, *P* = 0.002 for *S. serpillifolia*). RDA revealed no significant association between genotypes and environmental variables in *S. serpillifolia* (*P* = 0.08), but a significant correlation with geographical coordinates, which explained 11.4 % of the total genetic variation. In *S. retusa*, RDA indicated significant effects of both geographical coordinates (*P* < 0.001) and environmental variables (*P* < 0.001), explaining 9.2 and 3.8 % of the variation, respectively.

## DISCUSSION

### Biogeographical pattern and refugial areas

*Salix retusa*, distributed over the whole European Alpine System, exhibits four genetic clusters corresponding to distinct geographical regions: (1) the Pyrenees, (2) the Apennines, (3) the Alps, and (4) the Carpathians and northern Balkan Peninsula (Fig. 2). While the Pyrenean cluster is the most isolated one, the other three clusters show admixture. Genetic patterns and high numbers of private alleles in the groups outside the Alps support the hypothesis of glacial refugial areas (Figs 1 and 2). The Balkan Peninsula and the Apennines are widely recognized for providing multiple refugial areas (Taberlet *et al*., 1998; Gentili *et al*., 2015; Spaniel and Resetnik, 2022). The evolutionary distinctiveness of the southern Apennines, supported by many high-mountain endemic plant species (Schmitt, 2017), is also reflected by the three unadmixed individuals from Maiella (Fig. 2A). A similar admixture pattern could also arise from a series of colonization events from the Alps towards the Apennines, accompanied by successive founder events. To confidently distinguish between these scenarios, demographic modelling would be necessary. Within the Alps, the absence of genetic clustering and the low number of private alleles, together with very similar genetic diversity measures, suggest a rapid and rather complete postglacial recolonization scenario from a refugial area from the Eastern Alps (groups 6 and 7). Admixture and gene flow among different mountain ranges highlight the remarkable dispersal capacity of *S. retusa*, underscoring its ability to migrate and establish populations across vast distances within relatively short timeframes. In contrast, the high number of private alleles and lower allelic richness in the distinct Pyrenean cluster support long-term isolation and glacial survival in this mountain range (Schmitt, 2017). Interestingly, all individuals of the Pyrenees grouped together, despite representing two different ploidy levels. These findings could indicate that *S. retusa* originated in the Pyrenees, which would also be supported by TreeMix and by the phylogenetic tree (Fig 2C; Supplementary Data Fig. S1). However, according to Ogutcen *et al*. (2024), the *S. retusa* complex diversified in preglacial times (11.33 Mya), which suggests our present data are inadequate to address the species’ origin.

Our study confirms that diploid *S. serpillifolia* is an endemic species of the Alps, consistent with the findings of Kosiński *et al*. (2017, 2019*b*). We identified two genetically distinct clusters within *S. serpillifolia* exhibiting an east–west differentiation and suggesting independent evolution in separate glacial refugia. Similar patterns have previously been found for various other organisms (Schönswetter *et al*., 2004; Schmitt, 2017). Tribsch and Schönswetter (2003) previously identified potential glacial refugia in the easternmost Alps for subalpine and alpine plant species. *Salix serpillifolia* occurs there locally with a distinct genetic group (10) and with a notably lower observed heterozygosity than the others. Additionally, the strikingly high number of private alleles (1348) compared to the western groups (2–8; Supplementary Data Table S10) suggests that this group may have survived glaciations in the classical eastern central refugial area (Tribsch and Schönswetter, 2003), where it accumulated private alleles due to geographical isolation. Subsequent rapid recolonization of the previously glaciated eastern parts of the Hohe Tauern (Ankogel and Goldberg Groups) may have resulted in a loss of genetic diversity in small founder populations. However, gene flow among *S. serpillifolia* groups was generally restricted, with admixture detected only in the Central Eastern Alps. Within the Western Alps, limited genetic differentiation and very similar genetic diversity measures suggest rapid postglacial recolonization from a single refugium. The f3-statistics and PCA support the hypothesis of two peripheral glacial refugia, west and east of the Alps, that served as sources for recolonization and secondary contact in the Southern and Central eastern Alps (group 9). This area has already been identified as a secondary contact zone for other *Salix* species (Pittet *et al*., 2025). Notably, regions containing central nunataks in Switzerland, as hypothesized by Kosiński *et al*. (2019*b*), and peripheral nunataks for calcareous species (Rota *et al*., 2024*a*), do not align with the uniform genetic structure of the western cluster observed in our study. Furthermore, the low number of private alleles does not fit the expectations of genetically distinct nunatak populations as observed in other genera (Schorr *et al*., 2012; Schönswetter and Schneeweiss, 2019). Consequently, our results do not support these areas as potential nunatak refugia for *S. serpillifolia*.

*Salix retusa* shows more lineages and potential refugial areas overall, which is consistent with its broader distribution in the European Alpine System. Contrary to our expectations, we found more distinct lineages and, therefore, more potential glacial refugia for *S. serpillifolia* than for *S. retusa* within the Alps. A weak genetic sub-structure across the Alps has also been observed in tetraploid *Ranunculus kuepferi*, whereas the diploid cytotype showed higher genetic partitioning (Cosendai *et al*., 2013). In this case, the asexual mode of reproduction in tetraploids explained their faster colonization of the Alps. However, both *S. retusa* and *S. serpillifolia* reproduce sexually and produce wind-dispersed seeds, facilitating efficient colonization. The polyploid *S. retusa* could have benefitted from larger and more protruding catkins, producing a larger number of seeds. According to Dullinger *et al*. (2012), *S. retusa* has a high carrying capacity (number of individual shoots), maximum seed yield and good germination rates compared to other alpine plants; data for *S. serpillifolia*, however, are missing. Based on our genetic data, we speculate that the higher ploidy of *S. retusa* did not improve its survival capacity during the last glaciations but probably facilitated the colonization of remote areas. In the near future, global warming and loss of suitable habitats might threaten alpine species, but the effects are species-specific, with endemics of the Alps more threatened by extinction (Dullinger *et al*., 2012). *Salix serpillifolia* is probably at a greater risk of extinction than *S. retusa.*

### Niche differentiation

Our results show that *S. retusa* has a wider ecological niche than *S. serpillifolia*, aligning with the hypothesis that polyploids have broader niches than their diploid ancestors (e.g. Levin, 1983; Otto and Whitton, 2000*b*). Within the Alps, we found that both species have highly similar niches but show different preferences for specific soil and topography conditions. This pattern supports the phylogenetic niche conservatism theory which suggests that closely related species exhibit greater ecological similarity than more distantly related species (Harvey and Pagel, 1991). Niche comparison indicates a small-scale displacement insofar as *S. retusa* expands its niche towards soils with lower organic carbon and higher clay content. Additionally, *S. serpillifolia* grows at higher elevations and on more south-facing slopes than *S. retusa*. We did not identify a clear adaptation of *S. retusa* to harsher climatic conditions. The polyploid niche only indicates a better adaptation to maximum temperature. This pattern is commonly associated with polyploidization (Van de Peer *et al*., 2020) and, in our case, could be related to the significantly larger leaves of *S. retusa* in comparison to *S. serpillifolia* (Kosiński *et al*., 2017). *Salix retusa* grows faster and is taller than *S. serpillifolia*, which may confer a greater competitiveness and allows *S. retusa* to occur at lower elevations, in warmer regions with higher vegetation. In contrast, *S. serpillifolia* is adapted to high elevations and wind-exposed habitats. Its more compact growth form is an adaptation to low temperatures (including freezing) and drought caused by strong wind exposure at higher elevations (e.g. Boucher *et al*., 2016). The characteristic small leaves of this species are also an adaptation to high elevations (Kosiński *et al*., 2017). Like other high-elevation plants, *S. serpillifolia* exhibits less discrimination against ^13^C uptake during photosynthesis than lowland species (the δ^13^C value is around −26 ‰ as in other alpine plants). Since δ^13^C in the atmosphere increases with elevation, high alpine plants can fix carbon more efficiently per leaf unit, and hence develop smaller leaves (Körner, 2021).

At the intraspecies level, we observed for both species a larger bioclimatic than edaphic niche displacement (Fig. 4). They can retain more similar edaphic and topographic preferences while adapting to varying climatic conditions. This finding suggests that soil requirements might reflect a shared physiological or evolutionary origin. However, it is also important to consider the limitations of the soil data used. Although we used global soil predictors at relatively high resolution, such data may still lack the spatial precision necessary to capture the fine-scale edaphic heterogeneity typical of alpine environments. In mountain regions, soil properties can vary sharply over short distances due to complex topography, diverse bedrock and microclimatic variation. As shown by Rota *et al*. (2024*b*), global soil datasets such as SoilGrids can underperform in such heterogeneous landscapes. Despite these limitations, Rota *et al*. (2024*b*) demonstrated that incorporating soil data can significantly improve the performance of models, particularly for species with strong edaphic preferences.

Nevertheless, our results for *S. retusa* suggest a broader edaphic tolerance in the Alps but a similar optimum compared to occurrences in other mountain regions. The niche of the Alpine occurrences demonstrates a higher flexibility, possibly correlated to the more heterogeneous habitats. Based on bioclimatic conditions, two different niches are observed outside the Alps (Fig. 4G). Occurrences in the Pyrenees, Apennines and Korab Massif share similar requirements, typical of sub-Mediterranean to Mediterranean high mountain climates. Occurrences in the Tatra Mountains, Rila Mountains, Šar Planina and South Carpathians fit with temperate climate conditions. Interestingly, this pattern reflects to some extent the intraspecific genetic structure and supports the idea that adaptation to local environmental conditions drives both genetic divergence and niche differentiation. Gene flow between adjacent, ecologically similar regions may maintain some level of genetic connectivity, while more distant populations are more ecologically and genetically distinct. These patterns highlight the interplay between historical processes, such as glaciation and isolation, and ecological factors in shaping both the genetic structure and the ecological niches of alpine species (Alvarez *et al*., 2009).

Concerning *S. serpillifolia*, the eastern range exhibits greater tolerance to low soil pH than the western range. Furthermore, it is restricted to growing sites with higher organic carbon content and a greater abundance of silicate minerals. As Hörandl *et al*. (2012) observed, *S. serpillifolia* occurs in the Central Alps (e.g. Hohe Tauern) on calcareous schists, which contains more silicate and has a lower pH than conditions found in the Western Alps. The niche differences between the two ranges of *S. serpillifolia* indicate local climatic adaptations, with better adaptation to greater temperature seasonality, higher total precipitation and higher temperature during the wet season in the Eastern Alps. Overall, our results show niche conservatism in the *S. retusa* complex with respect to edaphic and topographic conditions. However, concerning bioclimatic variables, niche optima are shifted at the intraspecific level, indicating strong ecological adaptation by different genetic lineages. In the *Senecio carniolicus* complex, niche differentiation between western and eastern diploids was larger to some extent than that between diploids and sympatric polyploids (Sonnleitner *et al*., 2016). Our findings highlight the ecological flexibility of polyploids compared to their diploid relatives. The restricted gene flow between the two lineages of *S. serpillifolia*, coupled with their substantial ecological differences, further supports the lower flexibility of the species. This outcome underscores the importance of considering intraspecific genetic structure to address species niche dynamics.

### Effects of polyploidy on genetic and ecological diversity

The greater genetic variation of *S. retusa* (Fig. 2A) may result from more ancient polymorphisms. Polyploids may retain ancient polymorphisms more effectively than their diploid relatives due to a larger effective population size, reducing the effects of genetic drift, thereby preserving allelic diversity over time (Husband *et al*., 2013). Furthermore, the buffering effect of multiple gene copies can lead to relaxed selection pressure and contribute to the retention of ancient genetic variation in polyploids (Otto and Whitton, 2000*a*). The evolutionary origin of *S. retusa* remains unknown; if it is of allopolyploid origin, as shown for most other polyploid willows (Wagner *et al*., 2020), the combined genomes from two or more unknown parental species could have established a higher genetic diversity. However, a similar genetic pattern across the range of *S. retusa* could also arise from local introgression with other willow species. Hybridization is common in *Salix* and has been reported even between genetically distantly related species (Argus, 1974; Gramlich *et al*., 2016, 2018). Based solely on morphological characteristics, *S. retusa* occasionally hybridizes with six other hexa- to octoploid willow species in nature (Wagner *et al*., 2021). All such hybrids are rare and morphologically distinguishable from the parental species, none of them were collected for this study. *Salix serpillifolia*, in contrast, hybridizes with two other diploid species in the Alps (Wagner *et al*., 2021). A preliminary phylogenetic network including all accessions and all potentially hybridizing species (not shown) did not show patterns of hybridization with other species. Moreover, due to the high ploidy level of *S. retusa*, hybridization with diploid species is considered unlikely as strong reproductive isolation is expected due to genetic incompatibilities (Husband and Sabara, 2004). If hybridization between *S. retusa* and diploid species were possible, we would expect to find *S. serpillifolia* × *S. retusa* hybrids in the Alps. To our knowledge, such hybrids have not been reported (Wagner *et al*., 2021). The substantial difference in ploidy between *S. retusa* and *S. serpillifolia* acts as a reproductive barrier and allows both species to occur in sympatry, even without large ecological niche displacement. However, small niche differences probably also contributed to a stable coexistence even if the competitive abilities of cytotypes differ (Mortier *et al*., 2024).

Our genetic findings also confirm those of Kosiński *et al*. (2019*a*), who concluded that *Salix kitaibeliana* Willd. is a synonym of *S. retusa.* The larger leaves of *S. retusa* found in the Tatra Mountains may be a response to higher temporal seasonality but cannot be assigned to a different lineage (Supplementary Data Table S19).

## CONCLUSION

Our study shows that both genetic patterns and niche differentiation have shaped the biogeographical pattern in the *S. retusa* polyploid complex, as has been shown generally by Rice *et al*. (2019) for polyploids. The hexa- to octoploid *S. retusa* benefits from a higher genetic diversity and a broader niche, which enabled its occupation of the entire European Alpine System. Diploid *S. serpillifolia* survived the LGM in peripheral glacial refugia within the easternmost Alps and successfully recolonized the Alps through adaptations to a distinct niche at high elevations and with extreme habitat conditions. Both niche differentiation within the Alps and the significant difference in ploidy levels are factors allowing sympatry of the two species in the Alps.

## Supplementary Material

mcaf163_Supplementary_Data

## Data Availability

Data supporting the findings are available within the manuscript and the Supplementary Data. All demultiplexed raw read data resulting from RAD-sequencing were submitted to the National Center for Biotechnology Information (NCBI) in the SequenceReadArchive under the BioProject ID 128 046. Sampling followed Nagoya regulations for the respective countries.

## SUPPLEMENTARY DATA

Fig. S1: Matrix of plots describing the statistics of soil and topography variables. Fig. S2: Matrix of plots describing the statistics of bioclimatic variables. Fig. S3: Evanno analysis plots for *Salix retusa*. Fig. S4: Genetic structure of *Salix retusa* for *K* = 3 to *K* = 8. Fig. S5: Likelihood and Delta m derived from TreeMix analysis for *Salix retusa*. Fig. S6: Evanno analysis plots for *Salix serpillifolia*. Fig. S7: Likelihood and Delta m values derived from TREEMIX analysis for *Salix serpillifolia*. Fig. S8: Contribution of soil and topography variables and bioclimatic variables to the first two PCs of the PCA. Fi. S9: Density plot for each environmental variable. Table S1: Name, description and abbreviation of the environmental layers considered. Table S2: Genetic statistics for the 12 groups of *Salix retusa*. Table S3: Pairwise *P*-values from tests comparing the rarefied allelic richness (Ark6) among groups of *Salix retusa*. Table S4: Pairwise *P*-values from tests comparing the number of effective alleles (NeA) among groups of *Salix retusa*. Table S5: Pairwise *P*-values from tests comparing the gene diversity (GD) among groups of *Salix retusa*. Table S6: Pairwise *P*-values from tests comparing the observed heterozygosity (HO) among groups of *Salix retusa*. Table S7: Pairwise *P*-values from tests comparing the number of private alleles (PA) among groups of *Salix retusa*. Table S8: Matrix of pairwise spatial and genetic distances for *Salix retusa*. Table S9: the f3-statistics in *Salix serpillifolia*. Table S10: Genetic statistics for the 10 groups of *Salix serpillifolia*. Table S11: Pairwise *P*-values from tests comparing the rarefied allelic richness (Ark6) among groups of *Salix serpillifolia*. Table S12: Pairwise *P*-values from tests comparing the number of effective alleles (NeA) among groups of *Salix serpillifolia*. Table S13: Pairwise *P*-values from tests comparing the gene diversity (GD) among groups of *Salix serpillifolia*. Table S14: Pairwise *P*-values from tests comparing the observed heterozygosity (HO) among groups of *Salix serpillifolia*. Table S15: Pairwise *P*-values from tests comparing the number of private alleles (PA) among groups of *Salix serpillifolia*. Table S16: Matrix of pairwise spatial and genetic distance for *Salix serpillifolia*. Table S17: Niche dynamics at the univariate level and tests of equivalency restricted to the soil and topographic conditions. Table S18: Model performance for soil and topography variables. Table S19: Niche dynamics at the univariate level and tests of equivalency restricted to the bioclimatic conditions. Table S20: Model performance for bioclimatic variables. Appendix S1: Methodology description of occurrence data extraction and filtering.
