# Supplementary material for: Niche expansion of polyploid cytotypes shaped the phylogeographical history of the Salix retusa complex in the European Alpine System
Source: Ann Bot. 2025 Jul 25;136(4):903–17. doi: 10.1093/aob/mcaf163 (PMC12464947; doi:10.1093/aob/mcaf163)
Supplement: mcaf163_Supplementary_Data [file mcaf163_supplementary_data.zip › Supplementary_Material_AOB_Pittet_2025.pdf]

Supplementary Material

Niche expansion of polyploid cytotypes shaped the phylogeographical history of the *Salix retusa* complex in the European Alpine System

Running title: Niche divergence in the alpine polyploid *Salix retusa* complex

Loïc Pittet[1,2*], Piotr Kosiński[3,4], Natascha D. Wagner[1], and Elvira Hörandl[1]

[1]Department of Systematics, Biodiversity, and Evolution of Plants (with Herbarium), University of Göttingen, Göttingen, Germany.

[2]Georg-August University School of Science (GAUSS), University of Göttingen, Göttingen, Germany.

[3]Faculty of Agriculture, Horticulture and Biotechnology, University of Life Sciences, Poznań, Poland.

[4]Institute of Dendrology, Polish Academy of Sciences, Kórnik, Poland.

Correspondence: Loïc Pittet, Department of Systematics, Biodiversity, and Evolution of Plants (with Herbarium), University of Göttingen, Germany. Email: lpittet@gwdg.de

The following Supporting Information is available for this article:

Figure S1: Matrix of plots describing the statistics of soil and topography variables.
Figure S2: Matrix of plots describing the statistics of bioclimatic variables.
Figure S3: Evanno analysis plots for *Salix retusa*.
Figure S4: Genetic structure of *Salix retusa* for K=3 to K=8.
Figure S5: Likelihood and Delta m derived from TREEMIX analysis for *Salix retusa*.
Figure S6: Evanno analysis plots for *Salix serpillifolia*.
Figure S7: Likelihood and Delta m values derived from TREEMIX analysis for *Salix serpillifolia.*
Figure S8: Contribution of soil and topography variables and bioclimatic variables to the first two PCs of the PCA.
Figure S9: Density plot for each environmental variable.
Table S1: Name, description and abbreviation of the environmental layers considered.
Table S2: Genetic statistics for the 12 groups of *Salix retusa*.
Table S3: Pairwise p-values from tests comparing the rarefied allelic richness (Ark6) among groups of *Salix retusa.*
Table S4: Pairwise p-values from tests comparing the number of effective alleles (NeA) among groups of *Salix retusa.*
Table S5: Pairwise p-values from tests comparing the gene diversity (GD) among groups of *Salix retusa.*
Table S6: Pairwise p-values from tests comparing the observed heterozygosity (HO) among groups of *Salix retusa.*
Table S7: Pairwise p-values from tests comparing the number of private alleles (PA) among groups of *Salix retusa.*
Table S8: Matrix of pairwise spatial and genetic distance for *Salix retusa*.
Table S9: F3-statistics in *Salix serpillifolia*.
Table S10: Genetic statistics for the 10 groups of *Salix serpillifolia.*
Table S11: Pairwise p-values from tests comparing the rarefied allelic richness (Ark6) among groups of *Salix serpillifolia.*
Table S12: Pairwise p-values from tests comparing the number of effective alleles (NeA) among groups of *Salix serpillifolia.*
Table S13: Pairwise p-values from tests comparing the gene diversity (GD) among groups of *Salix serpillifolia.*
Table S14: Pairwise p-values from tests comparing the observed heterozygosity (HO) among groups of *Salix serpillifolia.*
Table S15: Pairwise p-values from tests comparing the number of private alleles (PA) among groups of *Salix serpillifolia.*

Table S16: Matrix of pairwise spatial and genetic distance for *Salix serpillifolia*.
Table S17: Niche dynamics at the univariate level and tests of equivalency restricted to the soil and topographic conditions.
Table S18: Model performance for soil and topography variables.
Table S19: Niche dynamics at the univariate level and tests of equivalency restricted to the bioclimatic conditions.
Table S20: Model performance for bioclimatic variables.
Appendix S1: Methodology description of occurrences data extraction and filtering.

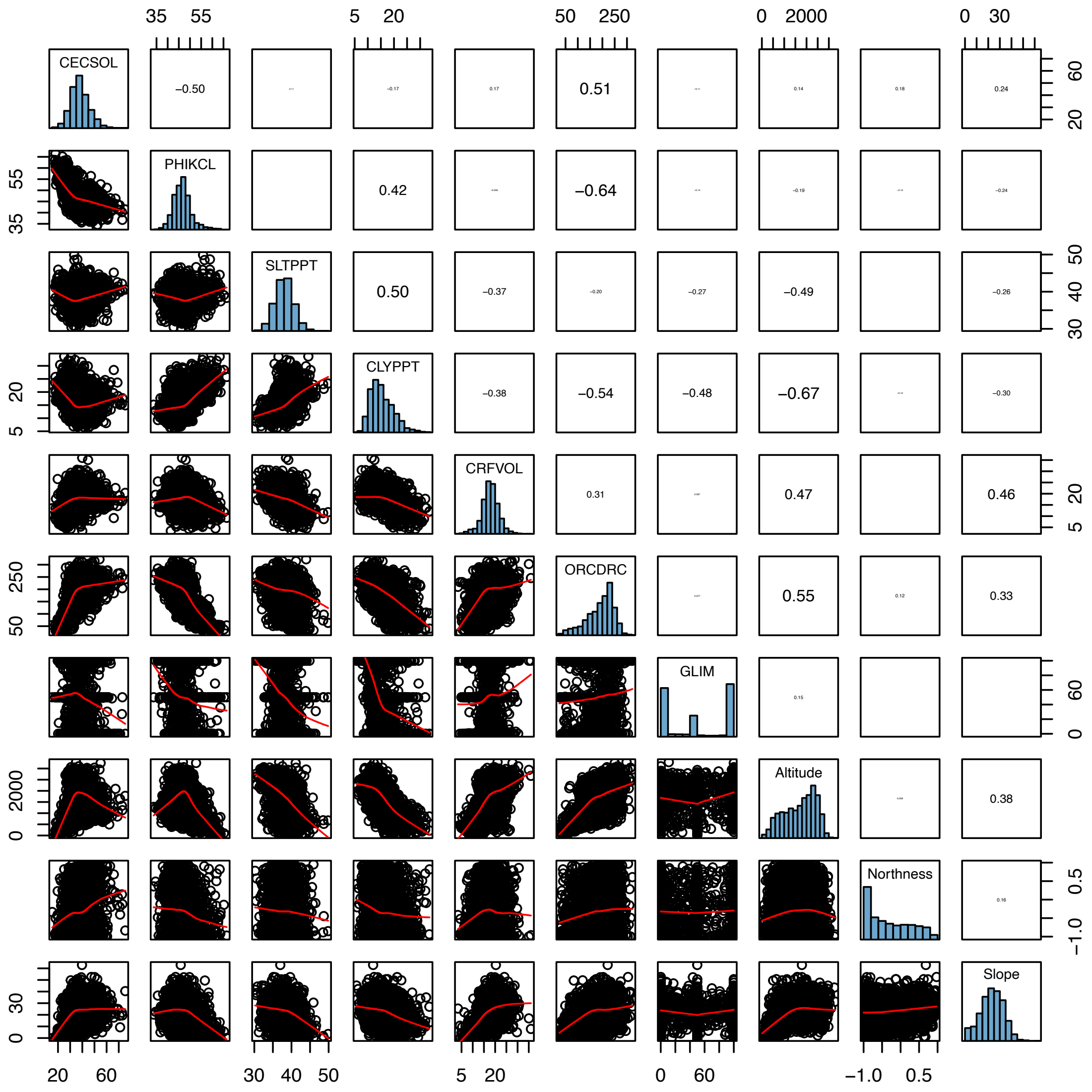

Figure S1: Matrix of plots describing the statistics of soil and topography variables. Bivariate scatter plots are shown below the parallel, histograms of each variable on the diagonal and Pearson correlation between pairs of variables above the diagonal. Abbreviations: CECSOL = Cation exchange capacity, PHIKCL = pH in KCL, SLTPPT = Silt content, CLYPPT = Clay content, CRFVOL = Coarse fragments, ORCDRC = Organic carbon density, GLiM = Global Lithology Map aggregated. Variable sources are defined in Supporting Information Table **S1**.

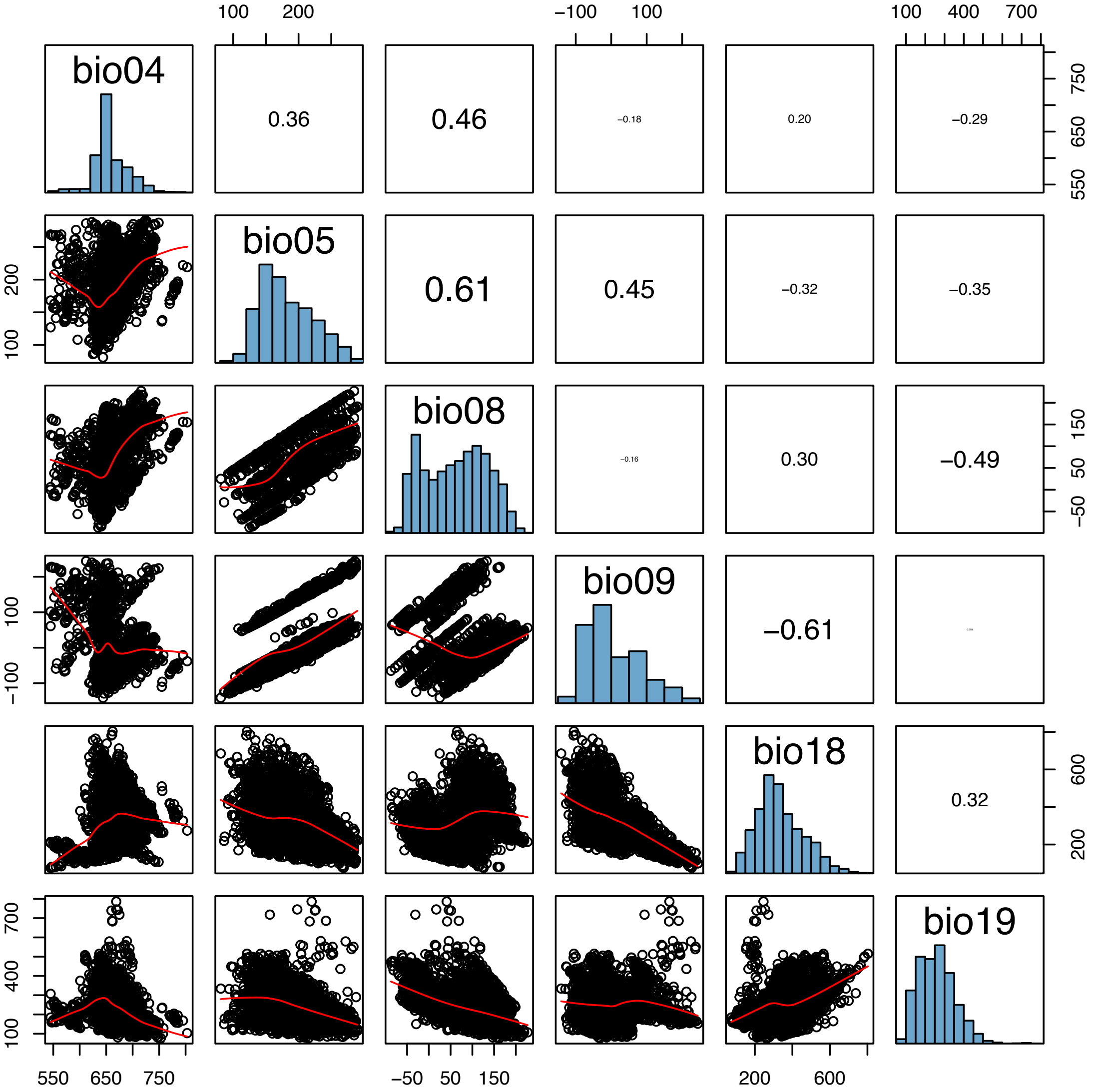

Figure S2: Matrix of plots describing the statistics of bioclimatic variables. Bivariate scatter plots are shown below the parallel, histograms of each variable on the diagonal and Pearson correlation between pairs of variables above the diagonal. Abbrevations: bio04 = Temperature seasonality, bio05 = Max temperature of warmest month, bio08 = Mean temperature of wettest quarter, bio09 = Mean temperature of driest quarter, bio18 = Precipitation of warmest quarter, bio19 = Precipitation of coldest quarter. Variable sources are defined in Supporting Information Table **S1**.

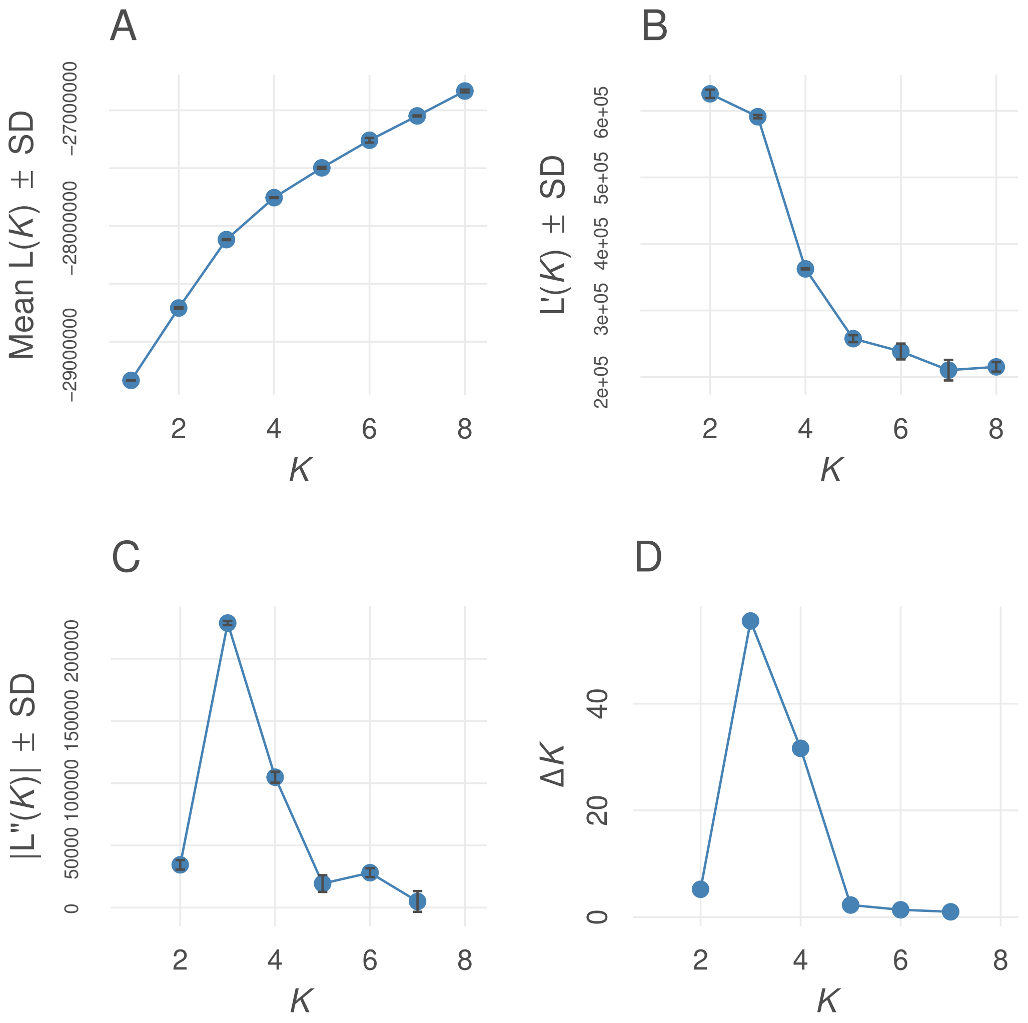

Figure S3: Evanno analysis plots for *Salix retusa*. (A) Estimated log probability of data over increasing values of K. (B) First derivate of the estimate log probability. (C) Second derivate of the estimate log probability. (D) Delta K over values of K.

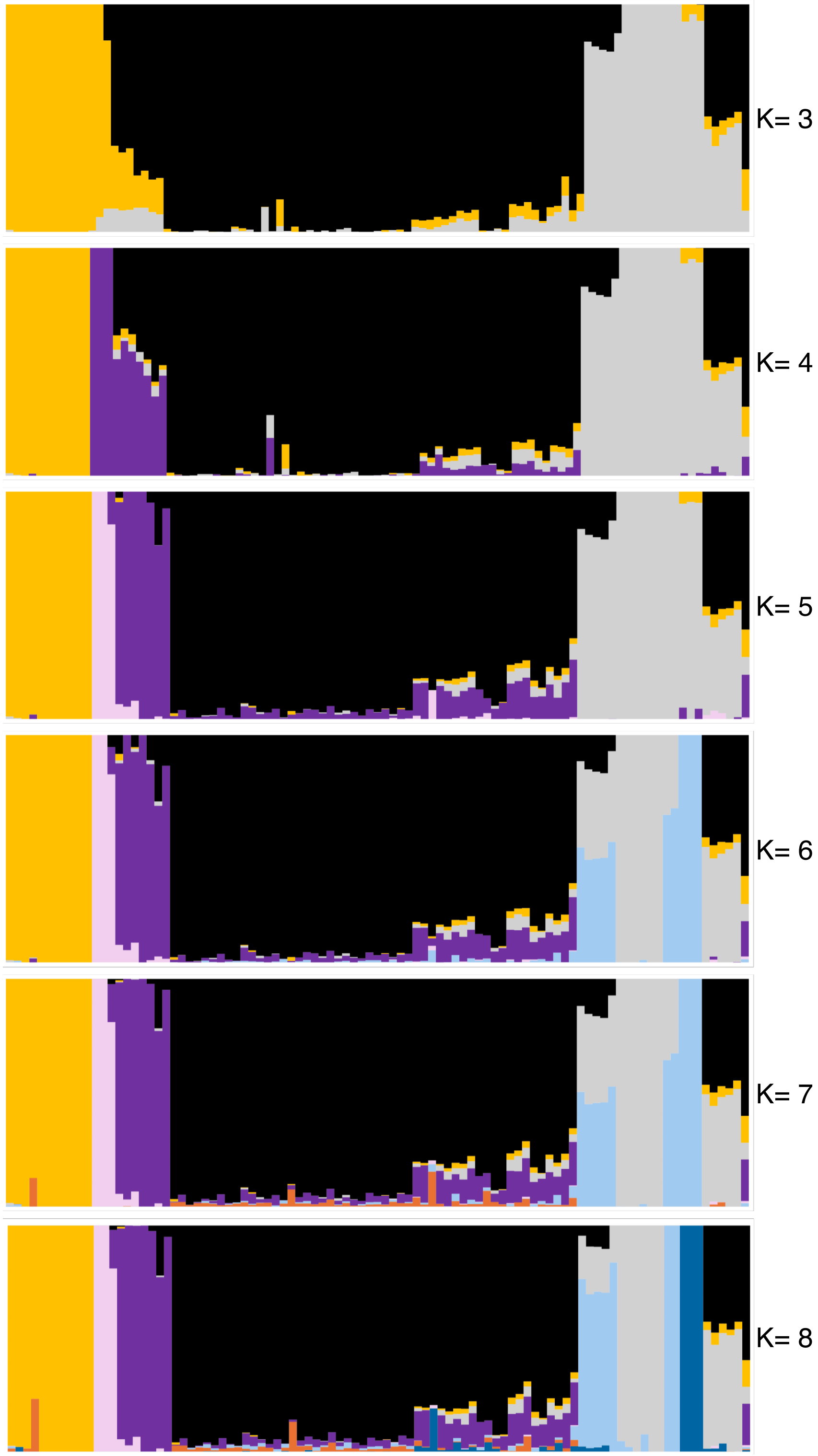

Figure S4: Genetic structure of *Salix retusa* across its whole distribution range for K=3 to K=8. The order is consistent with Fig. **2**.

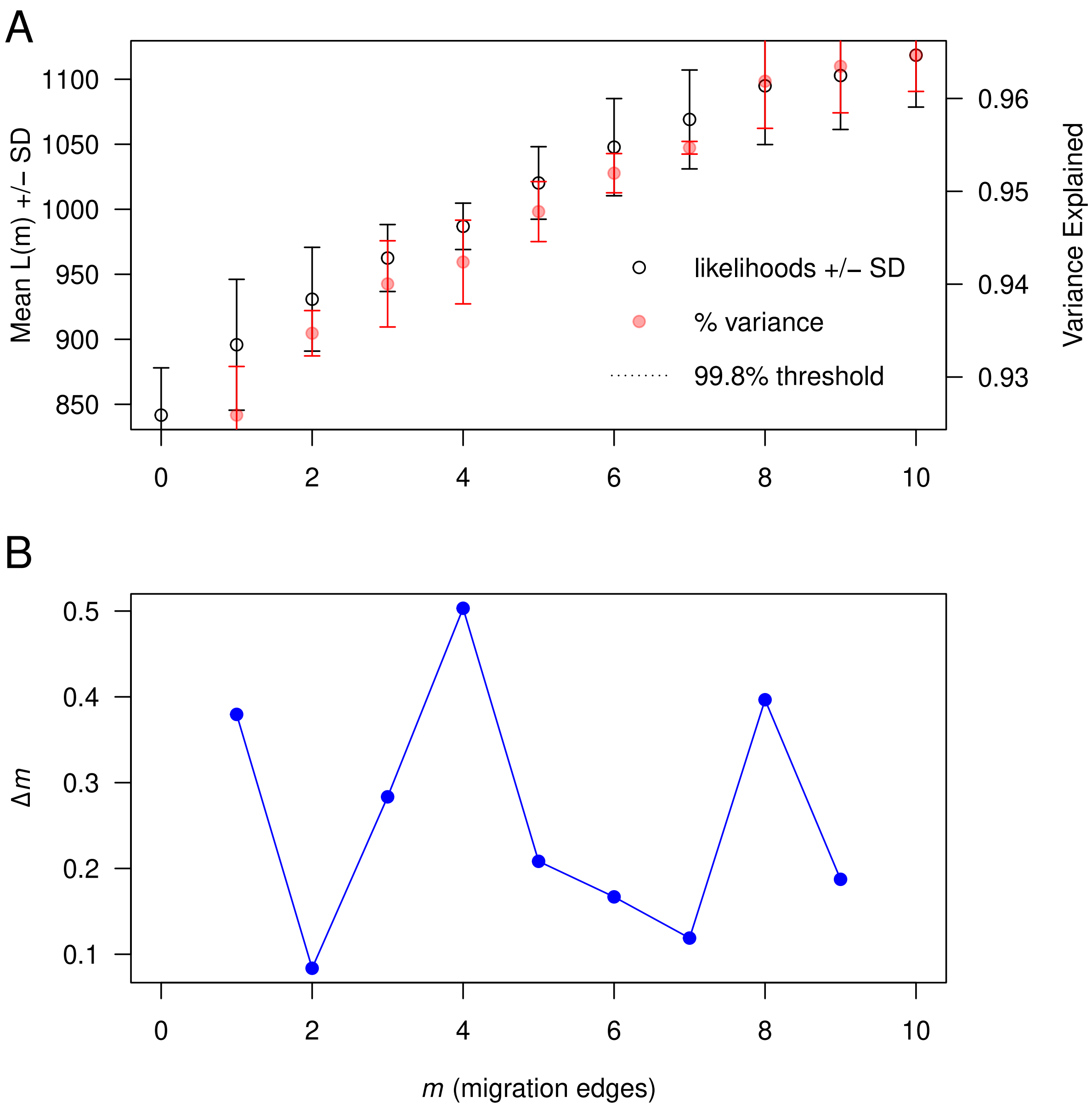

Figure S5: (A) Likelihood (white dot: mean and standard deviation) and variance explained (red dot: percentage). (B) Delta m values (blue dot) derived from TREEMIX analysis for *Salix retusa.*

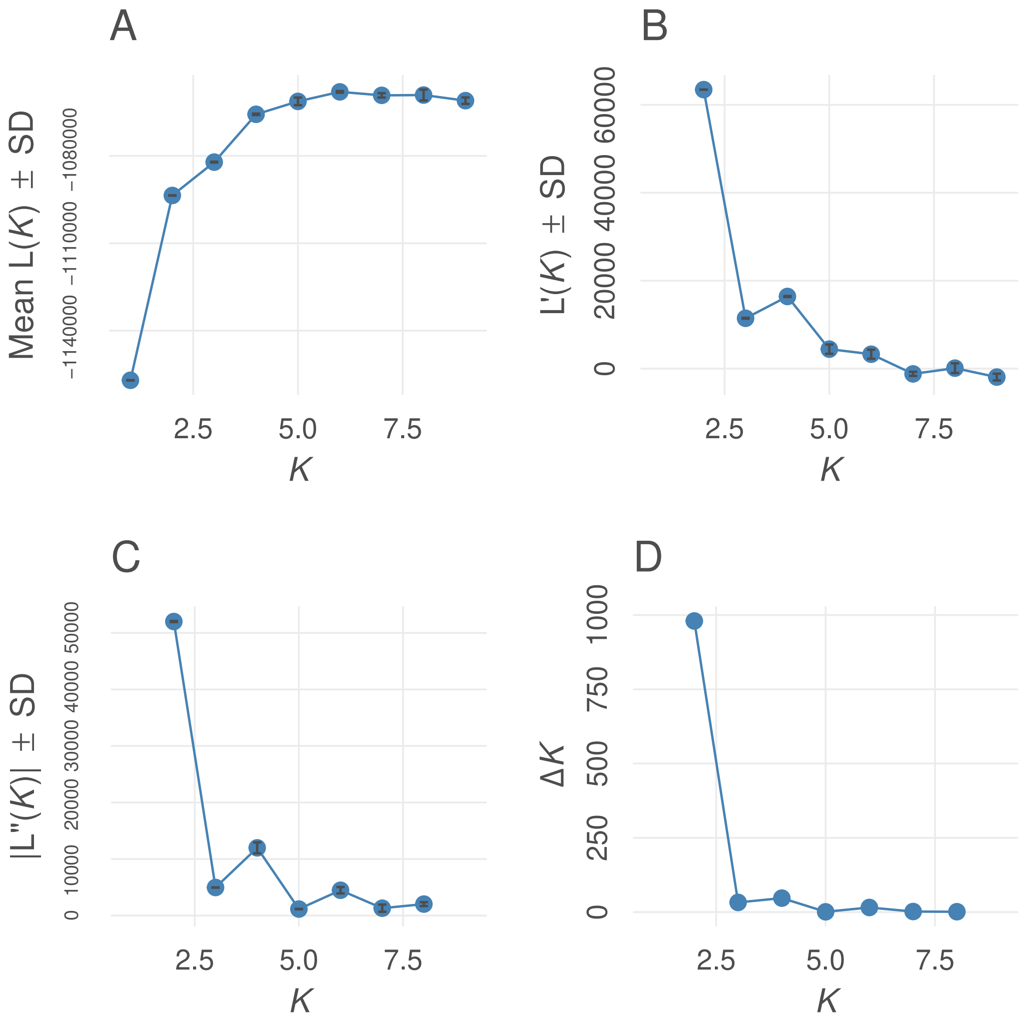

Figure S6: Evanno analysis plots for *Salix serpillifolia*. (A) Estimated log probability of data over increasing values of K. (B) First derivate of the estimate log probability. (C) Second derivate of the estimate log probability. (D) Delta K over values of K.

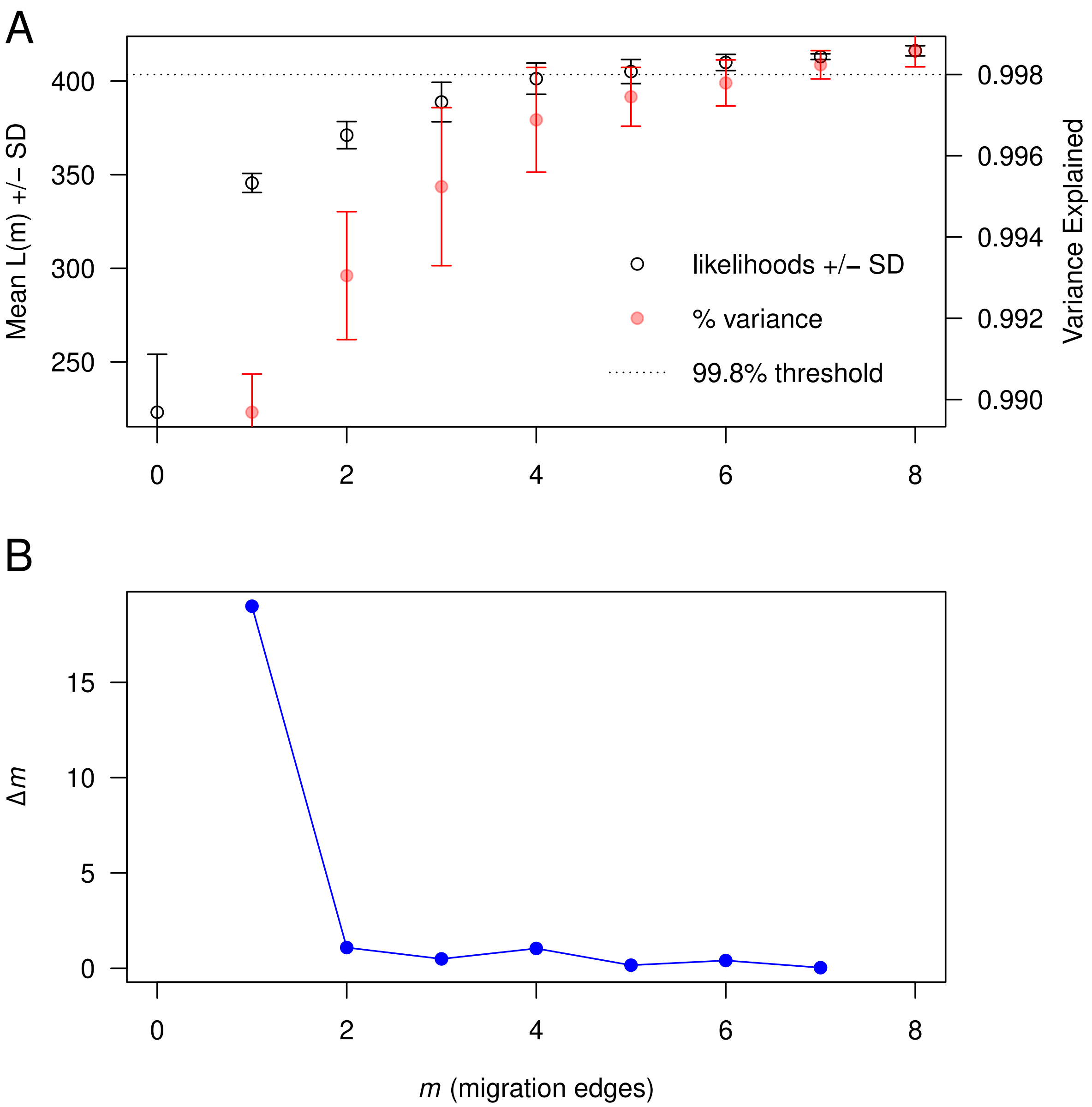

Figure S7: (A) Likelihood (white dot: mean and standard deviation) and variance explained (red dot: percentage). (B) Delta m values (blue dot) derived from TREEMIX analysis for *Salix serpillifolia.*

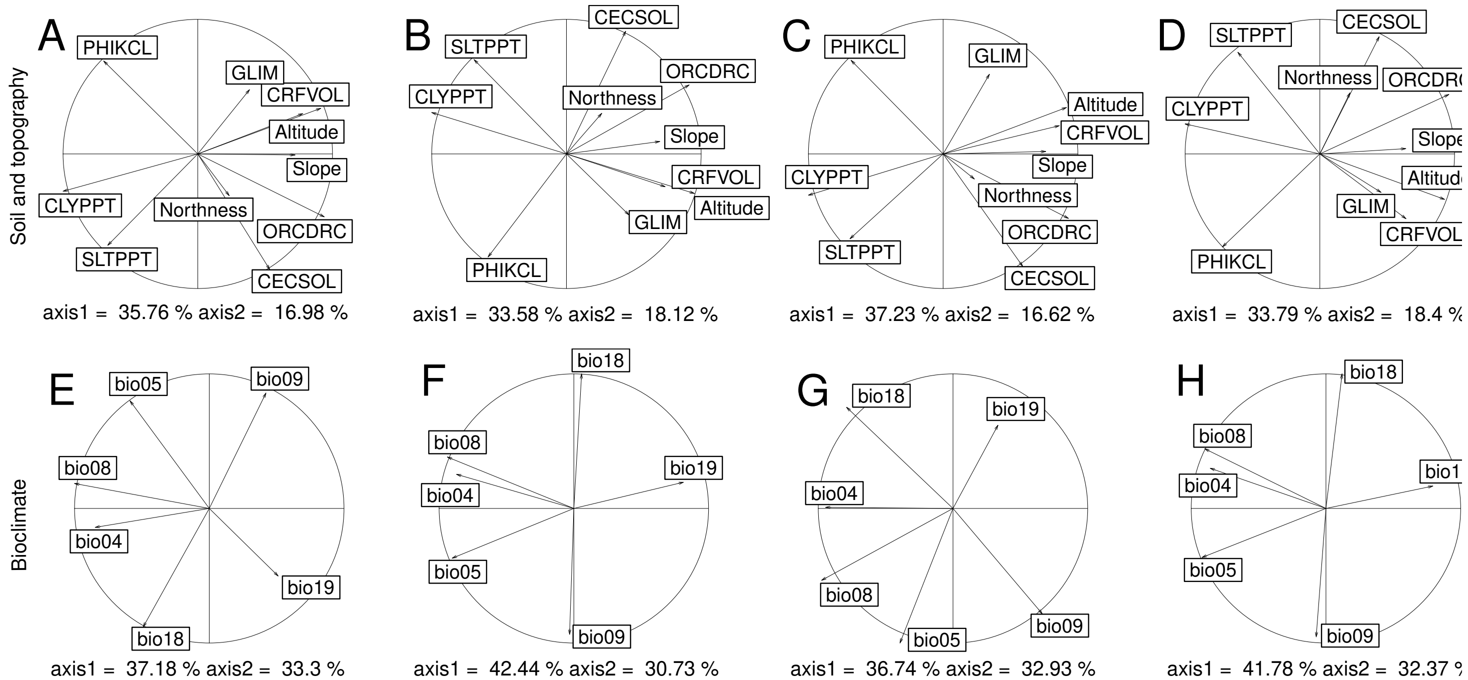

Figure S8: Contribution of soil and topography variables (A, B, C, D) and bioclimatic variables (E, F, G, H) to the first two PCs of the PCA between *Salix retusa* and *Salix serpillifolia* across their entire range (A, E), between *Salix retusa* and *Salix serpillifolia* in the Alps (B, F), between *Salix retusa* in the Alps and in other mountain ranges (C, G) and between *Salix serpillifolia* in the Western and Eastern Alps (D, H). Abbreviations: CECSOL = Cation exchange capacity, PHIKCL = pH in KCL, SLTPPT = Silt content, CLYPPT = Clay content, CRFVOL = Coarse fragments, ORCDRC = Organic carbon density, GLiM = Global Lithology Map aggregated, bio04 = Temperature seasonality, bio05 = Max temperature of warmest month, bio08 = Mean temperature of wettest quarter, bio09 = Mean temperature of driest quarter, bio18 = Precipitation of warmest quarter, bio19 = Precipitation of coldest quarter. Variable sources are defined in Supporting Information Table **S1.**

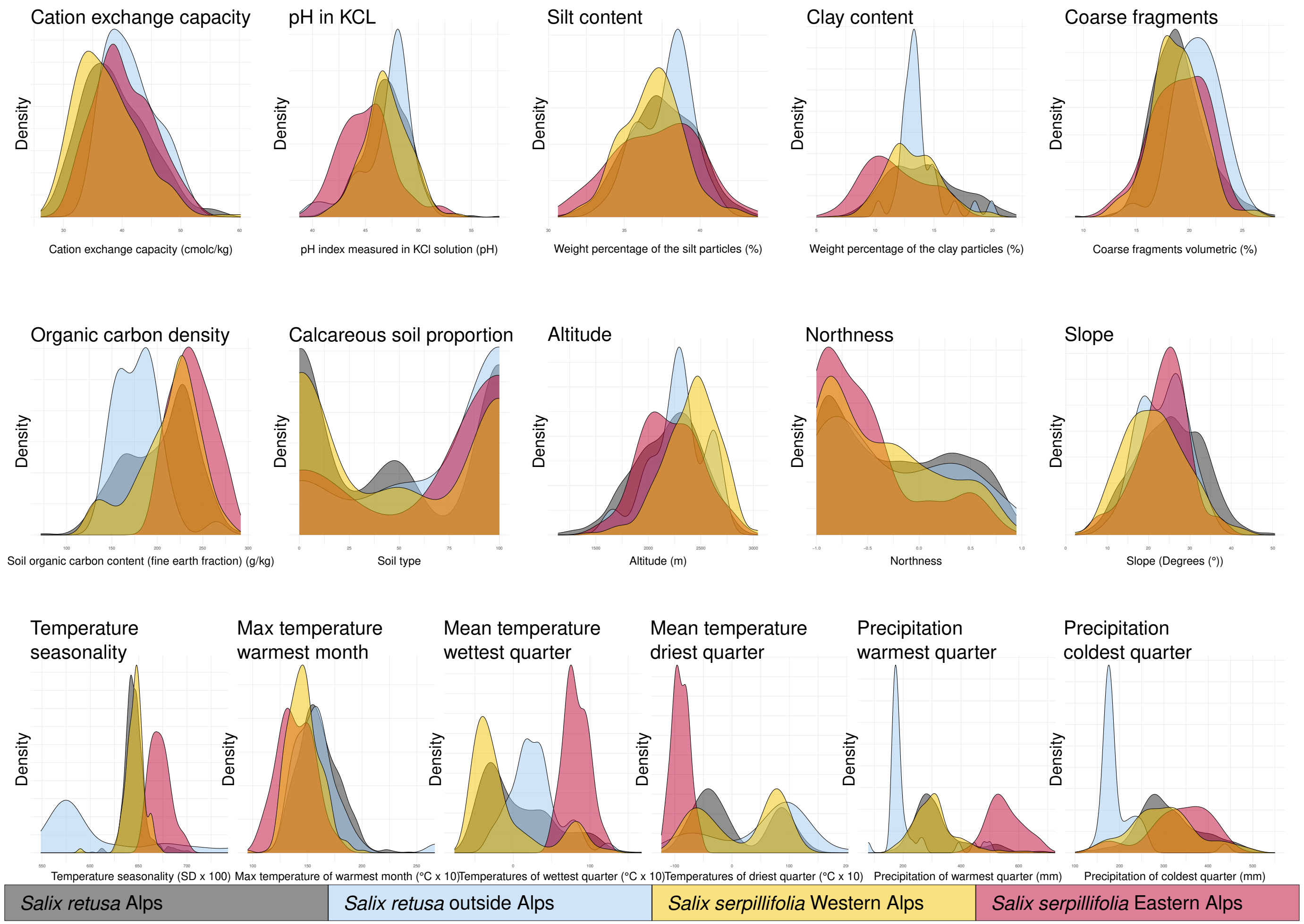

Figure S9: Density plot for each variable for *Salix retusa* in the Alps (gray), *Salix retusa* outside the Alps (blue), *Salix serpillifolia* in the Western Alps (yellow) and the *Salix serpillifolia* in the Eastern Alps (red).

Table S1: Name, description and abbreviation of the environmental layers considered.

Name	Set	Abbreviation	Included
GLiM: global lithological map aggregated	Soil and topography	GLIM	Yes
SoilGrids: Cation exchange capacity	Soil and topography	CECSOL	Yes
SoilGrids: pH in KCL	Soil and topography	PHIKCL	Yes
SoilGrids: Silt content	Soil and topography	SLTPPT	Yes
SoilGrids: Clay content	Soil and topography	CLYPPT	Yes
SoilGrids: Coarse fragments	Soil and topography	CRFVOL	Yes
SoilGrids: Organic carbon density	Soil and topography	ORCDRC	Yes
SoilGrids: Available soil water capacity	Soil and topography	WWP	No
SoilGrids: Sand content	Soil and topography	SNDPPT	No
EU-DEM: Altitude	Soil and topography	ALTITUDE	Yes
EU-DEM: cosinus(Aspect)	Soil and topography	NORTHNESS	Yes
EU-DEM: Slope	Soil and topography	SLOPE	Yes
CHELSA: Annual mean temperature	Bioclimate	bio1	No
CHELSA: Mean diurnal range	Bioclimate	bio2	No
CHELSA: Isothermality	Bioclimate	bio3	No
CHELSA: Temperature seasonality	Bioclimate	bio4	Yes
CHELSA: Max temperature of warmest month	Bioclimate	bio5	Yes
CHELSA: Min temperature of coldest month	Bioclimate	bio6	No
CHELSA: Temperature annual range	Bioclimate	bio7	No
CHELSA: Mean temperature of wettest quarter	Bioclimate	bio8	Yes
CHELSA: Mean temperature of driest quarter	Bioclimate	bio9	Yes
CHELSA: Mean temperature of warmest quarter	Bioclimate	bio10	No
CHELSA: Mean temperature of coldest quarter	Bioclimate	bio11	No
CHELSA: Annual precipitation sum	Bioclimate	bio12	No
CHELSA: Precipitation of wettest month	Bioclimate	bio13	No
CHELSA: Precipitation of warmest month	Bioclimate	bio14	No
CHELSA: Precipitation seasonality	Bioclimate	bio15	No
CHELSA: Precipitation of wettest quarter	Bioclimate	bio16	No
CHELSA: Precipitation of driest quarter	Bioclimate	bio17	No
CHELSA: Precipitation of warmest quarter	Bioclimate	bio18	Yes
CHELSA: Precipitation of coldest quarter	Bioclimate	bio19	Yes

Table S2: Genetic statistics for the 12 groups of *Salix retusa.* DGC: Number of defined gene copies. NA: Number of alleles. NeA: Number of effective alleles. Ark6: Allelic richness expected among 6 gene copies. GD: Gene diversity corrected for sample size. HO: Observed heterozygosity. PA: Number of private alleles.

Group	Location	Sample Size	DGC	NA	NeA	Ark6	GD	HO	PA
1	Pyrenees	11	80.81	3.39	1.97	2.24	0.39	0.33	706
2	W. Alps	12	89.96	4.17	2.10	2.41	0.42	0.37	24
3	W. Alps	7	52.45	3.57	2.09	2.37	0.41	0.37	12
4	W. Alps	8	59.91	3.74	2.10	2.39	0.42	0.37	9
5	Alps	9	65.26	3.83	2.13	2.42	0.42	0.35	10
6	E. Alps	5	36.95	3.23	2.07	2.34	0.41	0.35	12
7	E. Alps	10	72.94	3.93	2.14	2.43	0.43	0.34	8
8	E. Alps	5	37.24	3.22	2.07	2.33	0.41	0.36	9
9	Carpathians Rila Mts.	11	82.32	3.98	2.24	2.49	0.44	0.36	38
10	Šar Planina Korab Massif	5	38.13	3.13	2.07	2.31	0.41	0.36	258
11	Tatra Mts.	5	37.22	3.18	2.14	2.36	0.42	0.37	41
12	Apennines	10	77.62	3.87	2.16	2.42	0.42	0.35	485

Table S3: Pairwise p-values from Wilcoxon rank-sum tests comparing the rarefied allelic richness (Ark6) among groups of *Salix retusa*. P-values were adjusted for multiple comparisons using the Bonferroni correction. **Bolded values** indicate statistically significant differences between pairs (adjusted $p < 0.01$).

	1	2	3	4	5	6	7	8	9	10	11
2	**0.00**										
3	**0.00**	**0.00**									
4	**0.00**	0.02	0.31								
5	**0.00**	1.00	**0.00**	**0.00**							
6	**0.00**	**0.00**	**0.00**	**0.00**	**0.00**						
7	**0.00**	**0.00**	**0.00**	**0.00**	1.00	**0.00**					
8	**0.00**	**0.00**	**0.00**	**0.00**	**0.00**	1.00	**0.00**				
9	**0.00**	**0.00**	**0.00**	**0.00**	**0.00**	**0.00**	**0.00**	**0.00**			
10	**0.00**	**0.00**	**0.00**	**0.00**	**0.00**	**0.00**	**0.00**	1.00	**0.00**		
11	**0.00**	**0.00**	1.00	**0.00**	**0.00**	0.05	**0.00**	**0.00**	**0.00**	**0.00**	
12	**0.00**	1.00	**0.00**	**0.00**	1.00	**0.00**	1.00	**0.00**	**0.00**	**0.00**	**0.00**

Table S4: Pairwise p-values from Wilcoxon rank-sum tests comparing the number of effective alleles (NeA) among groups of *Salix retusa*. P-values were adjusted for multiple comparisons using the Bonferroni correction. **Bolded values** indicate statistically significant differences between pairs (adjusted $p < 0.01$).

	1	2	3	4	5	6	7	8	9	10	11
2	**0.00**										
3	**0.00**	0.10									
4	**0.00**	1.00	1.00								
5	**0.00**	0.52	**0.00**	0.02							
6	**0.00**	**0.00**	0.30	**0.00**	**0.00**						
7	**0.00**	**0.00**	**0.00**	**0.00**	1.00	**0.00**					
8	**0.00**	**0.00**	**0.00**	**0.00**	**0.00**	1.00	**0.00**				
9	**0.00**	**0.00**	**0.00**	**0.00**	**0.00**	**0.00**	**0.00**	**0.00**			
10	**0.00**	**0.00**	0.02	**0.00**	**0.00**	1.00	**0.00**	1.00	**0.00**		
11	**0.00**	1.00	**0.00**	0.03	1.00	**0.00**	1.00	**0.00**	**0.00**	**0.00**	
12	**0.00**	**0.01**	**0.00**	**0.00**	1.00	**0.00**	1.00	**0.00**	**0.00**	**0.00**	1.00

Table S5: Pairwise p-values from Wilcoxon rank-sum tests comparing the gene diversity (GD) among groups of *Salix retusa*. P-values were adjusted for multiple comparisons using the Bonferroni correction. **Bolded values** indicate statistically significant differences between pairs (adjusted $p < 0.01$).

	1	2	3	4	5	6	7	8	9	10	11
2	**0.00**										
3	**0.00**	0.12									
4	**0.00**	1.00	1.00								
5	**0.00**	0.34	**0.00**	0.02							
6	**0.00**	**0.00**	1.00	**0.00**	**0.00**						
7	**0.00**	**0.00**	**0.00**	**0.00**	1.00	**0.00**					
8	**0.00**	**0.00**	**0.00**	**0.00**	**0.00**	1.00	**0.00**				
9	**0.00**	**0.00**	**0.00**	**0.00**	**0.00**	**0.00**	**0.00**	**0.00**			
10	**0.00**	**0.00**	0.07	**0.00**	**0.00**	1.00	**0.00**	1.00	**0.00**		
11	**0.00**	0.26	**0.00**	**0.00**	1.00	**0.00**	1.00	**0.00**	**0.00**	**0.00**	
12	**0.00**	**0.00**	**0.00**	**0.00**	1.00	**0.00**	1.00	**0.00**	**0.00**	**0.00**	1.00

Table S6: Pairwise p-values from Wilcoxon rank-sum tests comparing the observed heterozygosity (HO) among groups of *Salix retusa*. P-values were adjusted for multiple comparisons using the Bonferroni correction. **Bolded values** indicate statistically significant differences between pairs (adjusted $p < 0.01$).

	1	2	3	4	5	6	7	8	9	10	11
2	**0.00**										
3	**0.00**	1.00									
4	**0.00**	1.00	1.00								
5	**0.00**	**0.00**	**0.00**	**0.00**							
6	**0.00**	**0.00**	**0.00**	**0.00**	1.00						
7	**0.00**	**0.00**	**0.00**	**0.00**	**0.00**	**0.00**					
8	**0.00**	**0.00**	0.04	0.08	**0.00**	**0.00**	**0.00**				
9	**0.00**	0.06	0.40	0.85	**0.00**	**0.00**	**0.00**	1.00			
10	**0.00**	**0.00**	0.03	0.06	**0.00**	**0.00**	**0.00**	1.00	1.00		
11	**0.00**	**0.00**	**0.00**	**0.00**	**0.00**	**0.00**	**0.00**	**0.00**	**0.00**	**0.00**	
12	**0.00**	**0.00**	**0.00**	**0.00**	0.02	**0.00**	**0.00**	**0.00**	**0.00**	**0.00**	**0.00**

Table S7: Pairwise p-values from Wilcoxon rank-sum tests comparing the number of private alleles (PA) among groups of *Salix retusa*. P-values were adjusted for multiple comparisons using the Bonferroni correction. **Bolded values** indicate statistically significant differences between pairs (adjusted $p < 0.01$).

	1	2	3	4	5	6	7	8	9	10	11
2	**0.00**										
3	**0.00**	0.16									
4	**0.00**	0.16	1.00								
5	**0.00**	0.34	1.00	1.00							
6	**0.00**	**0.00**	1.00	1.00	1.00						
7	**0.00**	0.07	1.00	1.00	1.00	1.00					
8	**0.00**	**0.00**	1.00	1.00	1.00	1.00	1.00				
9	**0.00**	**0.00**	**0.00**	**0.00**	**0.00**	**0.00**	**0.00**	**0.00**			
10	**0.00**	1.00	**0.00**	**0.00**	**0.01**	**0.00**	**0.00**	**0.00**	**0.00**		
11	**0.00**	1.00	0.03	0.03	0.06	**0.00**	**0.01**	**0.00**	**0.00**	1.00	
12	**0.00**	**0.00**	**0.00**	**0.00**	**0.00**	**0.00**	**0.00**	**0.00**	**0.00**	**0.00**	**0.00**

Table S8: Matrix of pairwise spatial distance (above diagonal) and pairwise genetic distance (Fst/(1-Fst); below diagonal) for the 12 groups of *Salix retusa.*

	1	2	5	3	4	7	6	8	11	9	10	12
1		6.805	7.004	5.913	7.159	11.507	11.298	13.704	19.983	23.049	19.442	12.435
2	0.060		0.212	1.252	0.740	4.734	4.518	7.000	13.219	16.796	13.691	6.919
5	0.058	0.002		1.368	0.606	4.549	4.331	6.826	13.032	16.654	13.585	6.851
3	0.061	0.001	0.000		1.281	5.832	5.608	8.137	14.285	18.017	14.943	8.158
4	0.060	0.000	0.000	0.001		4.568	4.343	6.885	13.008	16.833	13.882	7.254
7	0.055	0.004	0.003	0.002	0.001		0.228	2.329	8.486	12.412	9.921	4.607
6	0.056	0.004	0.001	0.004	0.003	0.004		2.557	8.702	12.639	10.126	4.705
8	0.055	0.004	0.001	0.004	0.003	0.003	0.002		6.305	10.125	7.954	4.322
11	0.099	0.043	0.035	0.043	0.041	0.034	0.040	0.038		6.235	7.331	9.350
9	0.095	0.046	0.044	0.044	0.044	0.040	0.038	0.034	0.014		4.404	10.743
10	0.077	0.030	0.032	0.031	0.030	0.020	0.024	0.025	0.049	0.032		7.015
12	0.069	0.032	0.030	0.031	0.030	0.024	0.025	0.021	0.055	0.055	0.049	

Table S9: F3-statistics in *Salix serpillifolia*. Significant negative values of f3 indicate that group A results from admixture between B and C. Only significant values are presented.

Test (A; B, C)	f3-statistic	Z-score
Group 9; Group 5, Group 10	-0.004	-11.74
Group 9; Group 6, Group 10	-0.004	-10.42
Group 9; Group 7, Group 10	-0.004	-11.32
Group 9; Group 3, Group 10	-0.004	-9.37
Group 9; Group 1, Group 10	-0.004	-9.19
Group 9; Group 4, Group 10	-0.004	-8.68
Group 9; Group 2, Group 10	-0.003	-8.33
Group 6; Group 2, Group 5	-0.001	-4.35
Group 9; Group 8, Group 10	-0.001	-3.08
Group 6; Group 1, Group 5	-0.001	-3.82
Group 6; Group 3, Group 5	-0.001	-2.81
Group 6; Group 4, Group 5	-0.001	-2.48
Group 6; Group 5, Group 7	-0.001	-2.03

Table S10: Genetic statistics for the 10 groups of *Salix serpillifolia*. DGC: Number of defined gene copies. NA: Number of alleles. NeA: Number of effective alleles. Ark4: Allelic richness expected among 6 gene copies. GD: Gene diversity corrected for sample size. HO: Observed heterozygosity. PA: Number of private alleles.

Group	Location	Sample Size	DGC	NA	NeA	ARk6	GD	HO	PA
1	Dauphiné Alps<br>Cottian Alps	5	9.9	1.61	1.4	1.23	0.230	0.222	1
2	Graian Alps	5	9.8	1.61	1.4	1.23	0.229	0.228	3
3	Lepontine Alps	6	11.7	1.61	1.38	1.22	0.220	0.209	32
4	Bernese Alps<br>Pennine Alps	4	7.9	1.58	1.4	1.23	0.231	0.228	0
5	Cottian Alps<br>Ligurian Alps	5	9.9	1.61	1.39	1.23	0.228	0.22	9
6	Maritime Alps<br>Cottian Alps	4	7.9	1.58	1.41	1.23	0.234	0.233	2
7	Glarus Alps<br>W. Rhaetian Alps<br>Bergam.Prealps	6	11.8	1.66	1.4	1.23	0.233	0.221	6
8	Ötztal Alps	4	7.9	1.53	1.39	1.22	0.221	0.241	21
9	Venetian Prealps<br>Dolomites	6	11.7	1.7	1.41	1.24	0.243	0.231	11
10	Western Tauern Alps<br>Seetal Alps	7	13.5	1.61	1.38	1.22	0.221	0.185	1348

Table S11: Pairwise p-values from Wilcoxon rank-sum tests comparing the rarefied allelic richness (Ark6) among groups of *Salix serpillifolia*. P-values were adjusted for multiple comparisons using the Bonferroni correction. **Bolded values** indicate statistically significant differences between pairs (adjusted $p < 0.01$).

	1	2	3	4	5	6	7	8	9
2	1.00								
3	**0.00**	**0.00**							
4	**0.00**	**0.00**	**0.00**						
5	1.00	1.00	**0.00**	**0.00**					
6	**0.00**	**0.00**	**0.00**	0.59	**0.00**				
7	1.00	1.00	**0.00**	0.87	1.00	**0.00**			
8	1.00	1.00	0.04	**0.00**	1.00	**0.00**	**0.00**		
9	**0.00**	**0.00**	**0.00**	**0.00**	**0.00**	0.14	**0.00**	**0.00**	
10	**0.00**	**0.00**	0.78	0.84	**0.00**	**0.00**	**0.00**	**0.00**	**0.00**

Table S12: Pairwise p-values from Wilcoxon rank-sum tests comparing the number of effective alleles (NeA) among groups of *Salix serpillifolia*. P-values were adjusted for multiple comparisons using the Bonferroni correction. **Bolded values** indicate statistically significant differences between pairs (adjusted $p < 0.01$).

	1	2	3	4	5	6	7	8	9
2	1.00								
3	**0.00**	**0.00**							
4	1.00	1.00	**0.00**						
5	1.00	1.00	**0.00**	1.00					
6	0.04	**0.00**	**0.00**	0.60	**0.00**				
7	1.00	1.00	**0.00**	**0.00**	1.00	1.00			
8	**0.00**	**0.00**	**0.00**	**0.00**	**0.00**	**0.00**	**0.00**		
9	**0.00**	**0.00**	**0.00**	**0.00**	**0.00**	**0.00**	**0.00**	**0.00**	
10	**0.00**	0.01	1.00	**0.00**	0.07	1.00	**0.00**	**0.00**	**0.00**

Table S13: Pairwise p-values from Wilcoxon rank-sum tests comparing the gene diversity (GD) among groups of S*alix serpillifolia*. P-values were adjusted for multiple comparisons using the Bonferroni correction. **Bolded values** indicate statistically significant differences between pairs (adjusted $p < 0.01$).

	1	2	3	4	5	6	7	8	9
2	1.00								
3	**0.00**	**0.00**							
4	**0.00**	**0.00**	**0.00**						
5	1.00	1.00	**0.00**	**0.00**					
6	**0.00**	**0.00**	**0.00**	0.59	**0.00**				
7	**0.00**	0.05	**0.00**	0.47	0.25	**0.00**			
8	1.00	1.00	**0.01**	**0.00**	1.00	**0.00**	**0.00**		
9	0.02	**0.00**	**0.00**	**0.00**	**0.00**	0.25	**0.00**	**0.00**	
10	**0.00**	**0.00**	0.05	1.00	**0.00**	**0.00**	**0.00**	**0.00**	**0.00**

Table S14: Pairwise p-values from Wilcoxon rank-sum tests comparing the observed heterozygosity (HO) among groups of *Salix serpillifolia*. P-values were adjusted for multiple comparisons using the Bonferroni correction. **Bolded values** indicate statistically significant differences between pairs (adjusted $p < 0.01$).

	1	2	3	4	5	6	7	8	9
2	0.16								
3	**0.00**	**0.00**							
4	**0.00**	**0.00**	**0.00**						
5	1.00	**0.00**	**0.00**	**0.00**					
6	**0.00**	**0.00**	**0.00**	0.32	**0.00**				
7	**0.00**	**0.00**	**0.00**	**0.01**	**0.00**	1.00			
8	0.06	1.00	0.73	0.51	**0.00**	**0.00**	**0.00**		
9	1.00	1.00	**0.00**	**0.00**	1.00	**0.00**	**0.00**	**0.00**	
10	**0.00**	**0.00**	**0.00**	**0.00**	**0.00**	**0.00**	**0.00**	**0.00**	**0.00**

Table S15: Pairwise p-values from Wilcoxon rank-sum tests comparing the number of private alleles (PA) among groups of *Salix serpillifolia*. P-values were adjusted for multiple comparisons using the Bonferroni correction. **Bolded values** indicate statistically significant differences between pairs (adjusted $p < 0.01$).

	1	2	3	4	5	6	7	8	9
2	1.00								
3	**0.00**	**0.00**							
4	1.00	1.00	**0.00**						
5	0.51	1.00	0.01	0.12					
6	1.00	1.00	**0.00**	1.00	1.00				
7	1.00	1.00	**0.00**	0.64	1.00	1.00			
8	**0.00**	0.01	1.00	**0.00**	1.00	**0.00**	0.17		
9	0.17	1.00	0.06	0.04	1.00	0.56	1.00	1.00	
10	**0.00**	**0.00**	**0.00**	**0.00**	**0.00**	**0.00**	**0.00**	**0.00**	**0.00**

Table S16: Matrix of pairwise spatial distance (above diagonal) and pairwise genetic distance (Fst/(1-Fst); below diagonal) for the 10 groups of *Salix serpillifolia.*

	5	6	1	2	4	3	7	9	8	10
5		0.370	1.222	1.149	1.964	2.680	3.286	4.756	4.530	6.950
6	0.005		0.944	0.860	1.837	2.682	3.388	4.949	4.684	7.153
1	0.035	0.018		0.091	1.088	2.142	3.044	4.801	4.430	6.997
2	0.041	0.026	0.009		1.168	2.209	3.097	4.840	4.478	7.039
4	0.026	0.014	0.003	0.012		1.119	2.120	3.969	3.523	6.120
3	0.057	0.044	0.030	0.038	0.030		1.044	2.918	2.430	5.027
7	0.033	0.022	0.029	0.035	0.023	0.050		1.874	1.403	4.000
9	0.035	0.026	0.040	0.048	0.034	0.060	0.016		0.629	2.208
8	0.086	0.082	0.086	0.094	0.084	0.106	0.058	0.053		2.599
10	0.237	0.224	0.236	0.246	0.226	0.258	0.216	0.160	0.234	

Table S17: Niche dynamics at the univariate level and tests of equivalency restricted to the soil and topographic conditions. Exp, Sta and Unf represent expansion, stability and unfilling of the compared niches, respectively. Significant *P-values* after equivalency tests (p.Exp, p.Sta, p.Unf) are indicated in bold. Abbreviations: CECSOL = Cation exchange capacity, PHIKCL = pH in KCL, SLTPPT = Silt content, CLYPPT = Clay content, CRFVOL = Coarse fragments, ORCDRC = Organic carbon density, GLiM = Global Lithology Map aggregated. Variable sources are defined in Supporting Information Table **S1.**

S. retusa / *S. serpillifolia* (Alps)						
	Exp	Sta	Unf	p.Exp	p.Sta	p.Unf
CECSOL	0.01	0.99	0.01	0.2	0.2	0.24
PHIKCL	0.005	0.995	0	0.4	0.4	1
SLTPPT	0.01	0.99	0	0.17	0.17	1
CLYPPT	0.03	0.97	0	**0.02**	**0.02**	1
CRFVOL	0.01	0.99	0.01	0.33	0.33	0.22
ORCDRC	0.03	0.97	0	**0.02**	**0.02**	1
GLIM	0.03	0.97	0.01	0.1	0.1	0.1
ALTITUDE	0.02	0.98	0.01	**0.03**	**0.03**	0.3
NORTHN.	0.02	0.98	0	**0.04**	**0.04**	1
SLOPE	0.01	0.99	0.01	0.36	0.36	0.36
S. retusa (Alps / Pyrenees, Apennines, Korab Massif)						
	Exp	Sta	Unf	p.Exp	p.Sta	p.Unf
CECSOL	0.07	0.93	0	**0.01**	**0.01**	1
PHIKCL	0.13	0.87	0	**0.01**	**0.01**	**0.03**
SLTPPT	0.12	0.88	0	**0.01**	**0.01**	1
CLYPPT	0.07	0.93	0	**0.01**	**0.01**	1
CRFVOL	0.13	0.87	0	0.06	0.06	1
ORCDRC	0.02	0.98	0	**0.01**	**0.01**	1
GLIM	0.03	0.97	0	0.2	0.2	0.1
ALTITUDE	0.01	0.99	0.01	0.44	0.44	1
NORTHN.	0.01	0.99	0	0.21	0.21	1
SLOPE	0.01	0.99	0.01	0.47	0.47	1
S. retusa (Alps / Rila Mts., Tatra Mts., Carpathians)						
	Exp	Sta	Unf	p.Exp	p.Sta	p.Unf
CECSOL	0.15	0.85	0	**0.01**	**0.01**	1
PHIKCL	0.43	0.56	0.02	**0.01**	**0.01**	**0.02**
SLTPPT	0.01	0.99	0	0.88	0.88	1
CLYPPT	0.01	0.99	0	0.91	0.91	1
CRFVOL	0.07	0.93	0	0.1	0.1	1
ORCDRC	0.04	0.96	0	0.2	0.2	1
GLIM	0.02	0.98	0.01	0.9	0.9	0.3
ALTITUDE	0.04	0.96	0.02	**0.01**	**0.01**	0.3
NORTHN.	0.04	0.96	0	0.27	0.27	1

SLOPE	0.01	0.99	0.03	0.96	0.96	**0.01**
			S. serpillifolia (Western Alps / Eastern Alps)			
	Exp	Sta	Unf	p.Exp	p.Sta	p.Unf
CECSOL	0.01	0.9	0	0.3	0.3	1
PHIKCL	0.05	0.95	0.03	0.6	0.6	**0.03**
SLTPPT	0.01	0.99	0	0.88	0.88	1
CLYPPT	0.05	0.95	0	0.5	0.5	1
CRFVOL	0.01	0.99	0.01	0.67	0.67	1
ORCDRC	0.32	0.67	0	**0**	**0**	1
GLIM	0.22	0.78	0.07	**0.04**	**0.04**	0.1
ALTITUDE	0.01	0.99	0	0.53	0.53	1
NORTHN.	0.14	0.86	0	0.07	0.07	1
SLOPE	0.04	0.96	0	0.5	0.5	1

Table S18: Model performance for soil and topography variables. Values in bold represent models significantly better than the null distribution.

		GAM	GLM	BC	DM	RF	MX
S. retusa	AUC	**0.89**	**0.85**	**0.79**	**0.65**	**0.98**	**0.9**
S. serpillifolia	AUC	**0.91**	**0.9**	**0.72**	**0.76**	**0.97**	**0.91**

Table S19: Niche dynamics at the univariate level and tests of equivalency restricted to the bioclimatic conditions. Exp, Sta and Unf represent expansion, stability and unfilling of the compared niches, respectively. Significant *P-values* after equivalency tests (p.Exp, p.Sta, p.Unf) are indicated in bold. Abbreviations: bio04 = Temperature seasonality, bio05 = Max temperature of warmest month, bio08 = Mean temperature of wettest quarter, bio09 = Mean temperature of driest quarter, bio18 = Precipitation of warmest quarter, bio19 = Precipitation of coldest quarter. Variable sources are defined in Supporting Information Table **S1.**

S. retusa / *S. serpillifolia* (Alps)						
	Exp	Sta	Unf	p.Exp	p.Sta	p.Unf
Bio04	0.01	0.99	0.01	0.63	0.63	1
Bio05	0.04	0.96	0.01	**0.01**	**0.01**	0.23
Bio08	0.01	0.99	0	0.35	0.35	1
Bio09	0.07	0.93	0.01	**0.02**	**0.02**	0.09
Bio18	0.01	0.99	0	0.45	0.45	1
Bio19	0.01	0.99	0	0.33	0.33	1
S. retusa (Alps / Pyrenees, Apennines, Korab Massif)						
	Exp	Sta	Unf	p.Exp	p.Sta	p.Unf
Bio04	1	0	1	**0.01**	**0.01**	**0.01**
Bio05	0.01	0.99	0.01	0.3	0.3	0.5
Bio08	0.15	0.85	0	**0.01**	**0.01**	1
Bio09	0	1	0.01	1	1	0.2
Bio18	0.1	0.9	0	**0.01**	**0.01**	1
Bio19	0.2	0.8	0	**0.01**	**0.01**	1
S. retusa (Alps / Rila Mts., Tatra Mts., Carpathians)						
	Exp	Sta	Unf	p.Exp	p.Sta	p.Unf
Bio04	0.8	0.2	0.62	**0.01**	**0.01**	**0.01**
Bio05	0.05	0.95	0	**0.02**	**0.02**	1
Bio08	0.47	0.53	0	**0.01**	**0.01**	1
Bio09	0.36	0.64	0	**0.01**	**0.01**	1
Bio18	0.04	0.96	0	0.45	0.45	1
Bio19	0.36	0.64	0.1	**0.01**	**0.01**	**0.01**
S. serpillifolia (Western Alps / Eastern Alps)						
	Exp	Sta	Unf	p.Exp	p.Sta	p.Unf
Bio04	0.45	0.55	0.41	**0.01**	**0.01**	**0.01**
Bio05	0.03	0.97	0.04	0.6	0.6	0.3
Bio08	0.7	0.3	0	**0.01**	**0.01**	1
Bio09	0.6	0.4	0	**0.01**	**0.01**	0.09
Bio18	0.9	0.1	0.03	**0.01**	**0.01**	**0.01**
Bio19	0.2	0.8	0	**0.01**	**0.01**	1

Table S20: Model performance for bioclimatic variables. Values in bold represent models significantly better than the null distribution.

		GAM	GLM	BC	DM	RF	MX
S. retusa	AUC	**0.92**	**0.86**	**0.87**	**0.86**	**0.98**	**0.92**
S. serpillifolia	AUC	**0.89**	**0.84**	**0.82**	**0.85**	**0.94**	**0.88**

Appendix **S1**: Methodology description of occurrences data extraction and filtering.

For both species, occurrence data were compiled from multiple sources, each of which was subjected to specific filtering procedures to ensure data quality.

EVA Database:
Vegetation relevés containing the target species were extracted from three EVA projects (IDs: 143, 186, and 237). The data were filtered to remove duplicate entries, records dated prior to 1980, observations with coordinate precision of fewer than four decimals, and records with spatial uncertainties exceeding 10 meters.

InfoFlora:
Raw occurrence data were obtained directly from InfoFlora. Filtering criteria included the removal of absence records, observations with uncertain taxonomic identification, introduced populations, imprecise geographic data (uncertainty exceeding 10 m), and records dated before 1980.

GBIF:
Occurrence data were also retrieved from the Global Biodiversity Information Facility (GBIF), following a modified version of the protocol described by Chauvier et al. (2021). We filtered data based on the "basis of record," retaining only entries classified as human observations, literature, material samples, general observations, and preserved specimens. Further filtering excluded duplicate records, entries prior to 1980, coordinates with fewer than four decimals, and spatial uncertainty exceeding 10 meters.

Personal Observations:
In addition to public databases, we incorporated personal field observations collected over the past 20 years.

The full, combined dataset was then plotted and visually inspected, and records considered spatially implausible were manually removed.
